# Indicators for Public Mental Health: A Scoping Review

**DOI:** 10.3389/fpubh.2021.714497

**Published:** 2021-09-27

**Authors:** Diana Peitz, Christina Kersjes, Julia Thom, Heike Hoelling, Elvira Mauz

**Affiliations:** Department of Epidemiology and Health Monitoring, Robert Koch Institute, Berlin, Germany

**Keywords:** public mental health, surveillance, monitoring, indicator, scoping review

## Abstract

**Background:** To monitor population mental health, the identification of relevant indicators is pivotal. This scoping review provides a comprehensive overview of current indicators representing the various fields of public mental health core topics. It was conducted as a first step to build up a Mental Health Surveillance for Germany.

**Methods:** We conducted a systematic MEDLINE search via PubMed. This search was supplemented by an extensive examination of the websites of relevant national as well as international institutions in the context of public mental health and an additional internet search via Google. To structure the data, an expert-based focus group identified superordinate topics most relevant to public mental health to which the identified indicators could be assigned to. Finally, the indicator set was screened for duplicates and appropriate content to arrive at a final set.

**Results:** Within the various search strategies, we identified 13.811 records. Of these records, a total of 365 records were processed for indicator extraction. The extracted indicators were then assigned to 14 topics most relevant to public mental health as identified by the expert-based focus group. After the exclusion of duplicates and those indicators not meeting criteria of specificity and target group, the final set consisted of 192 indicators.

**Conclusion:** The presented indicator set provides guidance in the field of current concepts in public mental health monitoring. As a comprehensive compilation, it may serve as basis for future surveillance efforts, which can be adjusted and condensed depending on the particular monitoring focus. Our work provides insights into established indicators included in former surveillance work as well as recent, not yet included indicators reflecting current developments in the field. Since our compilation mainly concludes indicators related to mental health in adults, it should be complemented with indicators specific to children and adolescents. Furthermore, our review revealed that indicators on mental health promotion and prevention are underrepresented in current literature of public mental health and should hence be focused on within future research and surveillance.

## Introduction

One prior target emphasized by the World Health Organization (WHO) within the 2013th Mental Health Action Plan was that by the year 2020, “80% of countries will be routinely collecting and reporting at least a core set of mental health indicators every 2 years through their national health and social information systems […]” (1). Consecutively, all WHO member states were called to systematically gather, integrate, process, analyze, interpret, and regularly report data on the mental health of the population. These data should inform about the current state as well as trends of public mental health which may allow for evaluating measures taken in mental health prevention, promotion, and healthcare. According to that, results should serve as a reliable database for evidence-based policy advice to enable political stakeholders to plan, initiate, and assess necessary health political actions (1, 2). Most recently, COVID-19 pandemic alerted policy makers to the necessity of mental health surveillance, since public mental health appeared as vulnerable asset in need of protection and immediate crisis response (3).

Following WHO's suggestions, Germany's national public health institute, Robert Koch Institute (RKI), has begun to develop a concept for a Mental Health Surveillance (MHS) in Germany on behalf of the Federal Ministry of Health in 2019. The present study was conducted as a first step to build up a MHS for Germany. The effectivity and significance of such a surveillance approach depends on the careful selection of appropriate indicators, which sufficiently depict public mental health. To establish a solid basis for a future core indicator set, the project was started by gathering a broad range of reported indicators currently used in the field. To do so, one can draw on the work of several countries which pioneered in establishing indicator based mental health surveillance systems such as Canada (4, 5), Australia (6), or Switzerland (7). Apart from that, further suitable concepts resp. indicators for monitoring population mental health are under development as research on mental health progresses; thus, they might be found mainly in scientific papers. Nevertheless, there might be mental health related issues which hitherto are neither implemented in established surveillance systems nor addressed by current research. This calls for the inclusion of further sources such as routine or administrative data within the search strategy.

Therefore, designing a mental health surveillance system from the ground up, the purpose of the current scoping review was (1) to systematically identify important indicators in the field of public mental health on a population-based level using different search strategies, (2) to assign the hereby extracted indicators to important core topics regarding public mental health to structure this large body of data and thus (3) to identify currently most relevant topics in the field and possible gaps of indicators reflecting unattended domains in relation to public mental health.

## Materials and Methods

According to our purposes, we chose the methodology of a scoping review to depict a broad picture of the current state of knowledge. Arkey and O'Malley (8) provided a methodological framework for conducting a scoping review, which was specified by Levac et al. (9). The structure of the present work followed those recommendations and was furthermore orientated on the more recent PRISMA extension for Scoping Reviews (PRISMA-ScR) (10). Thus, the method section is itemized by (1) identifying the research questions, (2) identifying relevant studies, (3) study selection, and (4) charting the data, collating, summarizing, and reporting the results.

### Identifying the Research Question

Unlike a systematic review, a scoping review does not aim to evaluate the quality of the processed studies and/or reports. Instead, it informs on the extent of work already existing in the field and should help to recognize gaps within this research (8–10). Thus, it best meets our purpose of gaining a broad overview of available and utilized indicators in the field of public mental health in order to build a MHS for Germany from the ground up. Additionally, this approach serves to identify hitherto neglected areas in recent state-of-the-art research and monitoring work. Therefore, this scoping review deals with the following research question: **Which indicators on public mental health can be identified on the base of the current state of knowledge?**

#### Definition of an Indicator Within the Present Work

An ongoing surveillance system depends on indicators incorporating a clear title (1st level) and a clear definition resp. operationalization by explicit numerator and denominator concepts (2nd level) leading to an explicit database (3rd level), thus enabling the pursued comparisons over time (11). Within the here presented first step of indicator compilation, indicators were processed on title level (meaning theoretical concepts with empirical application on population level are referred to as indicators) to decide on their importance within the next step toward a final indicator set for a MHS.

### Identifying Relevant Studies

To meet the above-mentioned demands on depicting indicators regarding the broad field of public mental health, a comprehensive search strategy including various sources was used.

#### Data Sources

First, we conducted a systematic MEDLINE literature search via PubMed using the MIP (Method, Issue, Participants) schema (12).

Since public mental health inidcators are not solely reported in scientific publications available on databases as MEDLINE, we decided to enrich the systematic approach by additional strategies:

In order to include already established surveillance indicators we scanned the websites of international organizations [European Union (EU), WHO, and Organization for Economic Co-operation and Development (OECD)] and the public health institutes of the 35 member countries of the OECD. To identify the websites of these public health institutes, a member list of the “International Association of National Public Health Institutes” (which can be found here: www.ianphi.org) was utilized. Additional Google searches were conducted if the websites of a national public health institute were not registered in this list.

In order to include routine and administrative data as well as country-specific indicators relevant to mental health care in Germany we conducted a distinct search on the websites of selected national stakeholders of mental health care, such as professional associations, service providers and federal agencies (DRV/Deutsche Rentenversicherung, KBV/Kassenärztliche Bundesvereinigung, DGPPN/Deutsche Gesellschaft für Psychiatrie und Psychotherapie, Psychosomatik und Nervenheilkunde, BPtK/Bundespsychotherapeutenkammer, APK/Aktion Psychisch Kranke e.V., GKV Spitzenverband/Spitzenverband Bund der Krankenkassen, AOLG/Arbeitsgemeinschaft der Obersten Landesgesundheitsbehörden and Kleine Anfragen) and a key word search using Google for indicators of mental health care and mental health care research in Germany.

**Figure 1:**
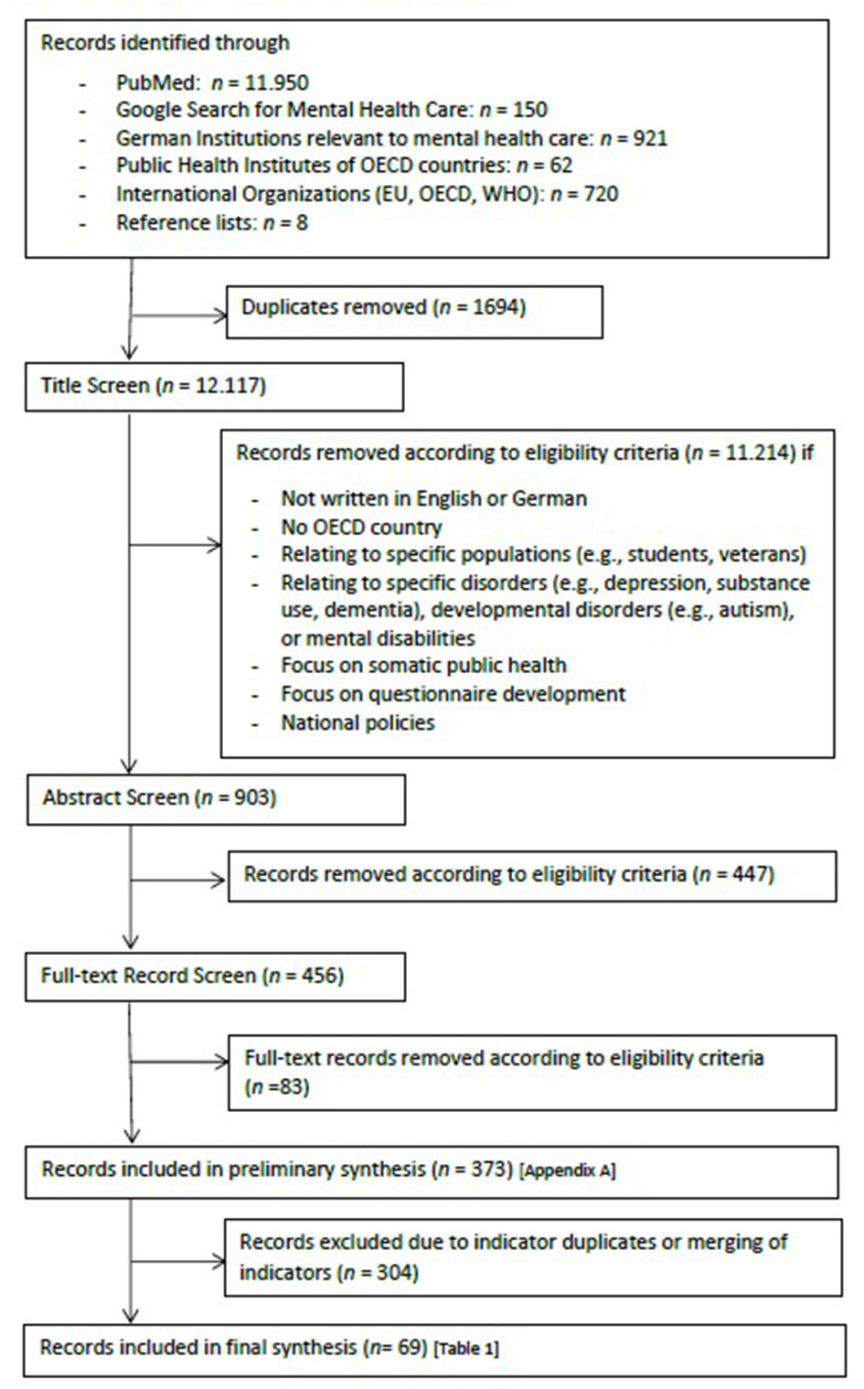
Flow chart scoping review.

Last, we scanned the reference lists of articles, which were found in the procedure mentioned above to identify further important information sources. The complete search was conducted from July to October 2019 and is depicted in Figure 1.

*DP and CK were in charge of the MEDLINE search while EM searched the websites of the public health institutes of different OECD countries and international organizations. JT conducted the particular search on mental health care indicators in Germany (administrative and routine data) and screened the reference lists*.

#### Key Words for Internet and Electronic Database Search

##### MEDLINE (via PubMed)

Broad research question regarding articles on surveillance/monitoring systems on mental health in the general population orientated on MIP schema:

(Surveillance [tiab] OR Surveillances [tiab] OR Monitoring [tiab] OR “Information system” [tiab] OR “Information systems” [tiab] OR “Population surveillance” [MeSH Terms] OR “Epidemiological Monitoring” [MeSH Terms]) AND (“mental health” [tiab] OR “mental-health” [tiab] OR “psychological health” [tiab] OR “mental disorder” [tiab] OR “mental disorders” [tiab] OR “mentally disordered” [tiab] OR “mental health problem” [tiab] OR “mental health problems” [tiab] OR “psychiatric disorder” [tiab] OR “psychiatric disorders” [tiab] OR “mental illness” [tiab] OR “mentally ill” [tiab] OR “mental sickness” [tiab] OR “mentally sick” [tiab] OR “mental disease” [tiab] OR “mental diseases” [tiab] OR “psychic health” [tiab] OR “psychiatric health” [tiab] OR “positive mental health” [tiab] OR “well-being” [tiab] OR “mental condition” [tiab] OR “psychological condition” [tiab] OR “mental constitution” [tiab] OR “mental health” [MeSH Terms] OR “mental disorders”[MeSH Terms]) AND (“general population” [tiab] OR “nationwide” [tiab] OR “nation-wide” [tiab] OR “representative survey” [tiab] OR “national sample” [tiab] OR “population-based” [tiab] OR “population based” [tiab] OR “Population wide” [tiab] OR “Population-wide” [tiab] OR “country wide” [tiab] OR “country-wide” [tiab] OR “representative sample” [tiab] OR national^*^ [tiab] OR “population level” [tiab]).

Specific research question regarding articles on applied indicators within surveillance/monitoring systems on mental health in the general population orientated on MIP schema:

(Indicator [tiab] OR Indicators [tiab] OR criteria [tiab] OR criterion [tiab] OR criterions [tiab] OR measure [tiab] OR measures [tiab] OR “Health status indicators” [MeSH Terms]) AND (Surveillance [tiab] OR Surveillances [tiab] OR Monitoring [tiab] OR “Information system” [tiab] OR “Information systems” [tiab] OR “Population surveillance” [MeSH Terms] OR “Epidemiological Monitoring” [MeSH Terms]) AND (“mental health” [tiab] OR “mental-health” [tiab] OR “psychological health” [tiab] OR “mental disorder” [tiab] OR “mental disorders” [tiab] OR “mentally disordered” [tiab] OR “mental health problem” [tiab] OR “mental health problems” [tiab] OR “psychiatric disorder” [tiab] OR “psychiatric disorders” [tiab] OR “mental illness” [tiab] OR “mentally ill” [tiab] OR “mental sickness” [tiab] OR “mentally sick” [tiab] OR “mental disease” [tiab] OR “mental diseases” [tiab] OR “psychic health” [tiab] OR “psychiatric health” [tiab] OR “positive mental health” [tiab] OR “well-being” [tiab] OR “mental condition” [tiab] OR “psychological condition” [tiab] OR “mental constitution” [tiab] OR “mental health” [MeSH Terms] OR “mental disorders”[MeSH Terms]).

##### Websites

- Selected national stakeholders of mental health care (e.g., professional associations, service providers, federal agencies): “Mental Disorders”
- International organizations (EU, WHO, OECD): “Mental Health Surveillance;” “Mental Health Indicators;” “Mental Health Monitoring”
- For each OECD country on www.ianphi.org: “Mental Health Surveillance;” “Mental Health Indicators;” “Mental Health Monitoring”

##### Google

- For each OCED listed public health institute **not** registered on www.ianphi.org “*[Country]* Mental Health Surveillance;” “*[Country]* Mental Health Indicators;” “*[Country]* Mental Health Monitoring”
- For relevant indicators on national public mental health care and mental health care research: “^*^Versorgungsforschung;” “psych^*^ Versorgung Bericht;” “psych^*^ Versorgungssituation;” “psych^*^ Versorgungsepidemiologie.” [translation: “^*^health care research;” “psych^*^ health care report;” “psych^*^ health care situation;” “psych^*^ epidemiology of health care.”]

### Study Selection

First, all records found via the several search paths were merged and checked for duplicates, which were then deleted. After that, documents were sequentially screened according to the inclusion and exclusion criteria defined below via the following steps: title screen, abstract screen, full record screen (see Figure 1).

We screened all types of published information (such as websites, brochures, posters, reports, scientific as well as official working and consultation papers) with a particular focus on indicators used to monitor mental health at a general population level in member states of the OECD. Records that met the following inclusion criteria were included: (1) availability in English or German language; (2) focus on public mental health; (3) focus on assessment at/database on population level (in contrast to e.g., clinical research or case studies); (4) current data (date of publication after 01.01.2000).

Since mental health surveillance needs to radically reduce complexity, only higher-order concepts can be addressed. Thus, records relating to databases on specific populations (e.g., students, veterans) instead of the general population or with a focus on developmental disorders (e.g., autism), mental disabilities, or specific mental disorders (e.g., depression, substance use, dementia) instead of broader psychopathology were excluded. Further, records with a focus on somatic public health, specific health behaviors as tobacco consumption or questionnaire development were excluded. Since indicators on national policies (existence: yes/no) do not allow for depicting changes at population-based level over time, such records were excluded as well.

*All screening steps were mainly done by three reviewers (DP, CK, and EM) who were supported by members of the Mental Health Research Unit within the Department of Epidemiology and Health Monitoring at RKI. The study group (DP, CK, and EM) met regularly to discuss challenges and uncertainties within the study selection process and to review and adjust inclusion and exclusion criteria in an iterative manner. Disagreement on study inclusion was reviewed in this group on a weekly base*.

### Charting and Collating of the Data

Charting and collating of the results required a conceptual framework, as the extensive area of public mental health needed to be structured substantially. For this purpose, the conceptual framework of an established part of the NCD surveillance at RKI [Diabetes Surveillance (13)] served as a grounded basis for a conducted expert-based focus group. This focus group consisted of nine members of the Mental Health Research Unit within the Department of Epidemiology and Health Monitoring at RKI who possessed a wide range of expert knowledge in various fields on public mental health and its monitoring (e.g., knowledge on health care, prevention & promotion, positive mental health, psychopathology, mental health across the lifespan, expertise in working with survey data or routine data, determinants of mental health). They consisted of psychologists, public health scientists, sociologists, methodologists, and trained psychotherapists. We used the technique of a focus group to pool this expert knowledge, to generate a wide range of different topics and to discuss discrepancies directly. The focus group was led by an experienced researcher (first author, DP) and took 125 min. The group was asked to collect the most important areas of public mental health surveillance, put them into writing and arrange them on a flip board; divergencies were discussed and the group itself agreed on the final topics. A comprehension of the discussion as well as the results were returned to the participants and approved by all of them.

The identified topics served as a grid to organize resulting data from each record that was considered as eligible for indicator extraction. To do so, a standardized data charting form was created in Microsoft Excel 2010 including the following information: authorship, publication year, indicator name, indicator definition (if available), assumed superordinate topic according to the focus group, whether the indicator was already contained in a surveillance resp. monitoring system and if it was specific to a certain age group.

On the basis of this data charting form, only mental health indicators on population-based level (see criteria for record inclusion above) were included. After a screen for duplicates, we excluded (1) indicators which were extracted from more than one record or subsumed similar content [e.g., “parental mental disorders” and “substance use (alcohol and drugs) by family members” as duplicates for “family history of mental disorders”]. Furthermore, we excluded (2) indicators within appropriate content for a continued population-based assessment (e.g., context of indicator too specific: “availability of a valued safe place where an individual can and wants to go to ‘escape' from things”) and (3) indicators, which showed non-specification for mental health surveillance (resp. relying on broader concepts, e.g., “general health”). Since the MHS for Germany was piloted for adults only, within this step (4) indicators for children and youth were excluded as well. To adequately identify indicators for this age group, several search steps would have had to be extended.

*Three reviewers (DP, CK, and EM) independently extracted indicators identified by the different search strategies with the help of the prepared data charting form. Uncertainties regarding the defined inclusion and exclusion criteria and consistency with the superior research question were discussed weekly. Afterwards, the assignment of the indicators to the single superordinate topics as well as the steps leading to the final set were aligned by the whole study group one by one*.

## Results

A total of 13.811 records were retrieved from the search. Figure 1 provides a detailed overview in which step and for what reason records were excluded. The various steps resulted in a total of 373 records which were processed for indicator extraction. A comprehensive list of these sources can be found in Appendix A.

### Important Topics for Public Mental Health

The conducted expert-based focus group led to the identification of 14 superordinate topics to cover the most relevant issues in terms of public mental health monitoring. The following topics were considered important:

1. Mental Health Promotion and Prevention
2. Mental Health Resources
3. Mental Health Risks
4. Mental Health Literacy
5. Positive Mental Health
6. Psychopathology
7. Self-harm and Suicidality
8. Supply and Utilization of Mental Health Care
9. Needs, Unmet Needs and Barriers in Mental Health Care
10. Quality of Care
11. Costs of Mental Disorders
12. Burden of Disease and Mortality
13. Participation
14. [Sociodemographic Variables with an Impact on Public Mental Health]

These topics built a preliminary conceptual outline which was used to collate the records and to roughly assign the indicators to.

### Indicator Assignment

In sum, *N* = 1.505 indicators could be extracted from the output generated by the literature research and were assigned to the superordinate topics. Screening for (1) duplicates (*n* = 920), (2) inappropriate content (*n* = 24), (3) non-specification for mental health surveillance (*n* = 31) and (4) indicators specified for children and youth (*n* = 349) resulted in a final set of 181 different indicators for the adult population. Table 1 depicts these indicators assigned to their superordinate topics.

**Table 1:** Indicators of public mental health in OECD countries.

**#**	**Indicator**	**Reference example**
**1. Mental Health Promotion and Prevention [*****n*** **=** **7 indicators]**		
1	*Mental health promotion budget*	(14)
2	Existence of mental health promotion programs	(15)
3	Presence of mental health promotion in schools	(14)
4	*Presence of programs to support parenting skills*	(16)
5	*Presence of suicide prevention programs*	(16)
6	*Participation in selected or indicated preventive programs on mental health*	(17)
7	*Anti-stigma movement*	(18)
**2. Mental Health Resources [*****n*** **=** **19 indicators]**		
8	Spirituality	(5)
9	Sleep	(19)
10	Healthy lifestyle [e.g., nutrition, physical activity, substance/alcohol consumption]	(20)
11	Self-efficacy	(21)
12	Resilience	(22)
13	Optimism	(7)
14	Personality	(19)
15	*General trust*	(20)
16	*Emotional intelligence*	(20)
17^*^	Self-esteem	(23)
18	Emotion regulation	(22)
19	Life-domain/Work-life balance	(7)
20	Satisfaction with work environment	(7)
21	Neighborhood environment	(20)
22	Perceived neighbourhood security	(20)
23	Sense of community belonging	(19)
24	Community involvement	(20)
25	Political participation	(20)
26	Social support/Social network	(5)
**3. Mental Health Risks [*****n*** **=** **16 indicators]**		
27	Chronic physical diseases	(19)
28	*Chronic pain*	(19)
29	Family history of mental disorders	(19)
30	*Family history of suicide-related behavior*	(19)
31	Critical life events trauma	(24)
32	Adverse childhood experiences	(24)
33	Violence	(20)
34	Discrimination	(20)
35	Chronic stress	(25)
36	High job strain	(7)
37^*^	Cognitive impairment	(26)
38	Housing conditions	(20)
39	Stressful neighborhood conditions	(20)
40	Income inequality in society	(27)
41	Homelessness	(28)
42	Loneliness	(29)
**4. Mental Health Literacy [*****n*** **=** **10 indicators]**		
43	Mental health-related knowledge	(28)
44^*^	Mental health locus of control	(30)
45	Attitudes towards mental disorders	(28)
46^*^	Attitude towards mental health services / Mental health care	(31)
47	Public attitudes toward people with a mental disorder	(32)
48	Social distance toward persons with mental disorders	(28)
49^*^	Perceived legitimacy of discrimination of persons with mental disorders	(33)
50^*^	Self-stigma	(31)
51^*^	Help-seeking attitudes	(31)
52	Competence in mental health self-management	(34)
**5. Positive Mental Health [*****n*** **=** **5 indicators]**		
53	Happiness	(28)
54	Health-related quality of life	(35)
55	Life satisfaction	(20)
56	Well-being [e.g., emotional/subjective well-being; psychological, social, physical]	(5)
57	*Meaning in life*	(22)
**6. Psychopathology [*****n*** **=** **34 indicators]**		
58	Prevalence of psychological distress	(36)
59^*^	*Prevalence of burnout*	(37)
60^*^	Incidence of any mental disorder (all F-diagnoses)	(38)
61	*Incidence of affective disorders*	(16)
62	*Incidence of anxiety disorders*	(16)
63^*^	*Incidence of substance use disorders*	(23)
64^*^	*Incidence of psychotic disorders*	(39)
65^*^	*Incidence personality disorders*	(38)
66	Prevalence of anxiety disorders	(36)
67^*^	Prevalence of mood/Affective disorders	(40)
68	Prevalence of depression	(29)
69	Prevalence of postpartum depression	(36)
70	Prevalence of bipolar disorders	(36)
71^*^	Prevalence manic episodes	(41)
72	Prevalence of alcohol use disorder	(42)
73	Prevalence of substance use disorder	(24)
74	*Prevalence of attention deficit hyperactivity disorders*	(43)
75	Prevalence of obsessive-compulsive disorder	(43)
76	Prevalence of schizophrenia	(43)
77^*^	Prevalence of psychotic disorder	(41)
78^*^	*Prevalence of adjustment disorder*	(41)
79	Prevalence of posttraumatic stress disorder	(43)
80	Prevalence of eating disorders	(43)
81^*^	Prevalence of impulse control disorders	(41)
82^*^	Prevalence of somatoform and dissociative disorders	(44)
83	Prevalence of personality disorders	(43)
84	Prevalence of sleep disorders	(19)
85	Prevalence of any mental disorder (all F-Diagnoses)	(22)
86	Prevalence of severe mental disorders	(43)
87	Prevalence of common/high prevalent mental disorders	(22)
88	Prevalence of depression and/or anxiety disorders	(45)
89	Prevalence of chronic mental disorders	(28)
90	Comorbidity physical disease	(22)
91	Comorbidity mental disorder	(46)
**7. Self-Harm and Suicidality [*****n*** **=** **6 indicators]**		
92	Self-harm	(19)
93	Suicidality [e.g., ideations, plans]	(19)
94	Suicide attempts	(19)
95	Suicide rate of the general population	(47)
96	Suicide rate of mental health inpatients and recently after discharge	(48)
97	PYLL due to suicide	(29)
**8. Supply and Utilization of Mental Health Care [*****n*** **=** **34 indicators]**		
98	Capacity of outpatient mental health care: mental health workers	(15)
99^*^	Capacity of outpatient mental health care: mental health specialists	(49)
100	Capacity of inpatient mental health care	(15)
101	Number of mental health hospitals	(15)
102	Number of psychiatric units in general hospitals	(15)
103	*Number of forensic inpatient units*	(15)
104	Number of mental health outpatient facilities attached to a hospital	(15)
105	Coverage of services for severe mental disorders	(50)
106	Treatment coverage for alcohol and drug dependence	(50)
107	Utilization of any health care of persons with diagnosed mental disorders^**^	(15)
108	Utilization of outpatient mental health care of persons with diagnosed mental disorders^**^	(15)
109	Utilization of primary health care of persons with diagnosed mental disorders for mental health reasons^**^	(29)
110	Utilization of primary health care of persons with diagnosed mental disorders for physical health reasons^**^	(51)
111^*^	Utilization of primary health care and somatic specialist care only of persons with diagnosed mental disorders^**^	(52)
112^*^	Proportion of psychotherapy in outpatient mental health care^**^	(53)
113^*^	Proportion of pharmacotherapy in outpatient mental health care [e.g., depression]^**^	(53)
114^*^	Treatment with psycho- and pharmacotherapy in outpatient care^**^	(54)
115	Utilization of inpatient care of persons with diagnosed mental disorders^**^	(34)
116^*^	Number of inpatient cases^**^	(55)
117	Number of days of inpatient stay^**^	(48)
118	Hospital discharges for mental disorders	(36)
119	*Number of long stay patients*	(17)
120	Inpatient readmissions by mental health diagnoses	(6)
121	Out-patient aftercare	(17)
122^*^	Utilization of psychiatric day care^**^	(56)
123	Utilization of home treatment^**^	(51)
124	Pre-admission community care	(6)
125	Emergency room visits	(19)
126	Self-help intervention utilization	(17)
127	Assisted housing for persons with mental disorders	(29)
128^*^	Utilization of rehabilitation measures due to mental disorders	(57)
129^*^	Number of days of rehabilitation measures due to mental disorders	(58)
130	Treatment with psychotropic drugs [e.g., antidepressants, antipsychotics, narcotic, sedative and anxiolytic substances]	(59)
131	*Opioid substitution treatment*	(60)
**9. Needs, Unmet Needs and Barriers in Mental Health Care**		
**[*****n*** **=** **12 indicators]**		
132^*^	Perceived needs	(61)
133^*^	Help-seeking behavior due to mental disorders	(62)
134^*^	*Treatment latency*	(63)
135	Early intervention [e.g., in psychosis]^**^	(51)
136^*^	Met mental health care needs for adults with mental disorders^**^	(64)
137	Unmet mental health care needs	(19)
138^*^	Number of cases per registered psychiatrist /psychotherapist	(56)
139	Waiting times ambulatory mental health care	(35)
140^*^	Waiting times inpatient mental health care	(65)
141^*^	*Waiting times psychiatric daycare*	(65)
142	*Waiting times in emergency rooms*	(32)
143^*^	Access barriers in mental health care	(66)
**10. Quality of Care [*****n*** **=** **13 indicators]**		
144	Patient satisfaction with mental health care system	(22)
145	Patient reported outcome measures	(51)
146	Treatment success	(17)
147^*^	Drop-out from mental health care	(67)
148	Patient education and participation	(17)
149^*^	*Inclusion of family and social environment into treatment of mental disorders*	(68)
150	Coercive measures: involuntary admission	(51)
151	Coercive measures: compulsory treatment	(60)
152	Coercive measures: seclusion	(22)
153^*^	Detection of depression in primary care	(69)
154^*^	Guideline adherence mental disorders with S3 health care guidelines	(53)
155^*^	*Transfer rate from primary to secondary care*	(52)
156^*^	*Outpatient-sensitive hospital cases*	(70)
**11. Costs due to Mental Disorders [*****n*** **=** **7 indicators]**		
157	Total expenditure on mental health services	(15)
158	Direct costs due to mental disorders	(71)
159^*^	Direct costs due to mental disorders - outpatient care	(72)
160^*^	*Direct costs due to mental disorders - rehabilitation*	(73)
161^*^	*Indirect costs due to mental disorders*	(74)
162	Sickness compensation	(16)
163	Disability pension for mental health reasons	(75)
**12. Burden of Disease and Mortality [*****n*** **=** **9 indicators]**		
164	Work loss due to mental health reasons	(28)
165	Functional Impairment due to mental health reasons	(59)
166	Mentally unhealthy days	(36)
167	DALYs (disability-adjusted life years)	(15)
168	YLDs (years lived with disability)	(24)
169	Excess mortality of people with mental disorders	(48)
170	Mortality for persons with severe psychiatric disorders	(76)
171	Alcohol-related deaths	(77)
172	Drug-related deaths	(77)
**13. Participation [*****n*** **=** **9 indicators]**		
173	Proportion of people with mental illnesses in employment	(32)
174	*Existence of employment programs for people with long-term mental problems*	(14)
175^*^	*Poverty among persons with mental illnesses*	(78)
176	Proportion of people in prison with mental illnesses	(22)
177	Social and political participation in people with mental illnesses	(22)
178	Housing situation of people with mental illness [e.g., homelessness]	(22)
179	Discrimination due to mental health problems	(22)
180^*^	*Stigma-related stress*	(33)
181^*^	Stigma coping	(33)
**14. Sociodemographic Variables with an impact on public mental**		
**health [*****n*** **=** **11 indicators]**		
182	Age	(79)
183	Gender	(79)
184	Region	(79)
185	Income equality / Social deprivation of the district (GINI)	(79)
186	Urbanization /Region	(79)
187	Migration background/Ethnicity	(79)
188	Relationship status (such as marriage)	(79)
189	Unemployment	(79)
190	Level of education	(79)
191	Income/ Poverty	(79)
192	Socio-economic status	(79)

*Indicator in Italics = indicator found only once*.

* *Indicator not included in an established surveillance system*.

** *Applicable to “any diagnosis of mental disorder” or itemized by different diagnoses*.

Of the 181 identified indicators, 134 stemmed from a national indicator system from at least one OECD country or an international organization. Forty-seven indicators were hitherto not included in an existing indicator system (Table 1; indicator marked with an ^*^).

In total, 146 indicators were found in two or more records, 35 indicators were only found once (Table 1; indicator marked in *italics*).

The indicators varied in their specification from being very specific (e.g., #141 “Waiting times in psychiatric daycare”) to very broad (e.g., #157 “Total expenditures on mental health services”). Some indicators were separated in sub-indicators (see # 150–152; three different indicators for coercive measures).

“Psychopathology” and “Supply and Utlilization of Mental Health Care” were the topics most indicators were assigned to while “Positive Mental Health” and “Mental Health Promotion and Prevention” were the topics with the fewest indicators.

Since sociodemographic variables such as age or gender were comprised in the majority of the screened literature in Appendix A, these variables were not counted resp. included in the above mentioned systematic but listed once. Instead of choosing a quantitative approach, we compared the once listed sociodemographic determinants with commonly recommended variables in global surveillance work (79) to stratify surveillance indicators, resulting in an additional set of *n* = 11 sociodemographic variables with an impact on public mental health.

## Discussion

Following WHO's call in monitoring the mental health of the population, this scoping review was conducted as a first step to build up an indicator-based surveillance system to observe the public mental health of the adult population in Germany on a regular base. Particularly, our present work informs about important topics and indicators in this field based on the current state of knowledge in Germany and other OECD countries.

Within the scoping review, we searched and processed all types of published information, including websites, brochures, posters, and reports to gather latest information in the field of public health, particularly on national level. This approach seems favorable to depict an exhaustive picture of important indicators in the field of public mental health based on the current state of knowledge in research, surveillance, and administrative/routine data on mental health care. Though, according to the source, indicators were very differently communicated, varying in their specification or whether an indicator could be divided in sub-indicators. Attunement regarding the broadness of scope and the consecutive operationalization should therefore be considered when processing the reported indicators for further monitoring work.

Current indicators on public mental health can be described as heterogonous as the field itself: They referred to a wide range of 14 important areas which are reflecting the mentioned topics and recommendations of the overall WHO framework (1, 79), not only with regard to mental disorders and their recovery, but also in terms of positive mental health and the field of mental health promotion and prevention. The need to sufficiently structure the broad field of mental health and chart the indicators accordingly was met by conducting an expert-based focus groups to identify these various superordinate topics.

Following the methodology of a scoping review, we included very different kinds of documents and sources. Therefore, we were able to include a wide range of indicators, developed and used in a variety of settings. For example, most of the indicators were extracted from established indicator-based surveillance systems on public mental health. This seems favorable as those indicators are likely to have been developed by an elaborate, expert-based consensus process and already been proven to predict public mental health within a monitoring system. Moreover, it can be assumed that there is an existing database for these indicators; this enables future monitoring work to compare own data with international data, such as indicators of different OECD countries. Moreover, a quarter of the extracted indicators showed to have strong significance regarding public mental health in scientific studies on the general population or routine/administrative data but are not part of any indicator system yet. Since our search approach was not limited to indicators from already existing indicator systems, it gives promising insights into these existing indicators, that reflect current issues or are of relevance in a particular part of public mental health, which otherwise might have been neglected. To build and adjust a new surveillance system from the ground up, this approach seems beneficial to identify specific and recently important concepts with appropriate indicators reflecting them.

Most of the indicators were found more than once resp. could be extracted from more than one data source. This gives a first indication regarding the emphasis and/or importance of these indicators in former surveillance or research work and thus for the mental health of the population. Furthermore, it shows that our literature search has mainly identified established concepts that are supported by multiple sources as relevant public mental health indicators. Though, the identification of hitherto less used indicators might direct to new trends in important fields (as for example stigma-related content, see indicator #180) and should be considered in building up new surveillance systems.

Most indicators were assigned to the topics “Psychopathology” and “Supply and Utilization of Mental Health Care.” On the contrary, our findings revealed that indicators on “Positive Mental Health” and particularly measures on “Mental Health Prevention and Promotion” were underrepresented regarding both the number of assigned indicators as well as their diversity of content. In terms of strengthening and promoting mental health at population level, a stronger focus on this area seems important in future monitoring work—particularly in light of the current COVID-19 pandemic and an immediate response to its impact.

### Limitations and Future Research

Since this scoping review was conducted as a first step in piloting a MHS for Germany, our comprehensive search was restricted regarding the following points:

It mainly focused on indicators for adults. Future research in the field should extend this compilation by searching and depicting indicators which reflect aspects of public mental health across the whole lifespan, including particular indicators for children and adolescents who differ in their age-specific risks and resources of mental health as well as mental disorders and their assessment. These should be searched explicitly with the help of extended search strings and inclusion of institutions and monitoring systems with a focus on this age group.

Since we were interested to depict characteristics of the German health care system in building up a national MHS, some of the indicators were specific to the German system like for example “Guideline adherence mental disorders with S3 health care guidelines” (#154) which is explicitly oriented to a national health care guideline. Thus, particularly indicators regarding mental health care based on administrative and routine data that require a specific data basis (e.g., the mapping of outpatient and inpatient care of the total population, which in Germany is divided into different data bodies of different service providers) should be checked carefully before processing. Further monitoring work has to consider their own national or international specifications to appropriately align the indicators with their system.

Moreover, our concentrated search on mental health care (to include information from administrative and routine data) might have led to an overemphasizing of these indicators in the extracted indicator compilation. However, research and practice in the field of public mental health generally rather concentrated on mental health care services, recovery, and rehabilitation than on prevention or promotion. Nevertheless, prioritizing mental health efforts on prevention and promotion of mental health as well as enhancing resources and positive mental health is demanded (1, 5, 79). Future efforts should thus focus on identifying and researching indicators to assess and monitor measures of population mental health promotion and prevention, even in early states of research.

Lastly, the focus group to structure the broad field of mental health consisted of experts that were not independent of each other, so there may have been influences from hierarchy or previous relationship. It was tried to counteract these effects by emphasizing the importance of the opinion of everyone, managing speaking times and initial individual brainstorming. However, the results were solely used to give a structure when charting the indicators. Our approach has shown to be beneficial, since all of these identified topics could be filled with indicators and could thus facilitate the appropriate communication of results.

## Conclusion

This scoping review presents a wide range of currently utilized indicators important to the broad field of public mental health. Our comprehensive compilation intended to serve as a solid basis on which future research and surveillance projects might build on. To do so, all or a selection of the identified indicators could be used and adjusted to the individual constitutional needs.

Furthermore, the introduced indicators might be supplemented or condensed depending on individual national or international demands and tailored to particular research questions or monitoring focus. As our compilation mostly reflects indicators related to mental health in adults, it should be extended with indicators specific to children's and adolescents' mental health to depict the whole lifespan.

## Data Availability Statement

The original contributions presented in the study are included in the article/Supplementary Material, further inquiries can be directed to the corresponding author.

## Author Contributions

EM, DP, JT, and CK conducted the literature search. EM, DP, and CK reviewed documents for inclusion, performed the data extraction, analyzed, and interpreted the data. DP provided methodological guidance with respect to the identification, selection of documents, to charting and reporting of results, and drafted the manuscript with substantial input from JT and EM. CK and DP compiled the attachments. CK did the final language proofreading. All authors contributed to the conception, design of the study, read, and approved the submitted manuscript.

## Funding

The MHS – Set-up of a National Mental Health Surveillance at Robert Koch Institute project has been funded by the Federal Ministry of Health (Grant Number: Chapter 1504 Title 54401).

## Conflict of Interest

The authors declare that the research was conducted in the absence of any commercial or financial relationships that could be construed as a potential conflict of interest.

## Publisher's Note

All claims expressed in this article are solely those of the authors and do not necessarily represent those of their affiliated organizations, or those of the publisher, the editors and the reviewers. Any product that may be evaluated in this article, or claim that may be made by its manufacturer, is not guaranteed or endorsed by the publisher.

## Abbreviations

AOLG: Arbeitsgemeinschaft der Obersten Landesgesundheitsbehörden
APK: Aktion Psychisch Kranke e. V.
BPtK: Bundespsychotherapeutenkammer
DGPPN: Deutsche Gesellschaft für Psychiatrie und Psychotherapie, Psychosomatik und Nervenheilkunde
DRV: Deutsche Rentenversicherung
EU: European Union
GKV Spitzenverband: Spitzenverband Bund der Krankenkassen
KBV: Kassenärztliche Bundesvereinigung
MHS: Mental Health Surveillance
OECD: Organization for Economic Co-operation and Development
RKI: Robert Koch Institute
WHO: World Health Organization.
