# Supplementary material for: Indicators for Public Mental Health: A Scoping Review
Source: Front Public Health. 2021 Sep 27;9:714497. doi: 10.3389/fpubh.2021.714497 (PMC8502920; doi:10.3389/fpubh.2021.714497)
Supplement: Supplementary file 1 [file Data_Sheet_1.docx]

**Appendix A**

**Total lists of records screened for indicator extraction**

1. Aldworth J, Colpe LJ, Gfroerer JC, Novak SP, Chromy JR, Barker PR, et al. The National Survey on Drug Use and Health Mental Health Surveillance Study: calibration analysis. International journal of methods in psychiatric research. 2010;19 Suppl 1:61-87.

2. Al-Windi A, Dag E, Kurt S. The influence of perceived well-being and reported symptoms on health care utilization: a population-based study. Journal of clinical epidemiology. 2002;55(1):60-6.

3. Amone-P'Olak K, Burger H, Huisman M, Oldehinkel AJ, Ormel J. Parental psychopathology and socioeconomic position predict adolescent offspring's mental health independently and do not interact: the TRAILS study. Journal of epidemiology and community health. 2011;65(1):57-63.

4. Andersson L, Nyman CS, Spak F, Hensing G. High incidence of disability pension with a psychiatric diagnosis in western Sweden. A population-based study from 1980 to 1998. Work (Reading, Mass). 2006;26(4):343-53.

5. Angermeyer MC, Dietrich S. Public beliefs about and attitudes towards people with mental illness: a review of population studies. Acta psychiatrica Scandinavica. 2006;113(3):163-79.

6. Angermeyer MC, Schomerus G. State of the art of population-based attitude research on mental health: a systematic review. Epidemiology and psychiatric sciences. 2017;26(3):252-64.

7. Arbeitsgruppe Psychiatrie der Obersten Landesgesundheitsbehörden. Psychiatrie in Deutschland. Strukturen, Leistungen, Perspektiven. 2007.

8. Australian Institute of Health and Welfare, editor. Mental health services—in brief. Canberra: AIHW2018.

9. Australian Institute of Health and Welfare. Mental health services in Australia: Key performance indicators for Australian Public Mental Health services. Canberra, Australia: Australian Government; 2018.

10. Australian Institute of Health and Welfare. Mental health services in Australia: NHA: Mental health-related indicators. Canberra, Australia: Australian Government; 2018.

11. Bansal N, Bhopal R, Netto G, Lyons D, Steiner MF, Sashidharan SP. Disparate patterns of hospitalisation reflect unmet needs and persistent ethnic inequalities in mental health care: the Scottish health and ethnicity linkage study. Ethnicity & health. 2014;19(2):217-39.

12. Bardach NS, Coker TR, Zima BT, Murphy JM, Knapp P, Richardson LP, et al. Common and costly hospitalizations for pediatric mental health disorders. Pediatrics. 2014;133(4):602-9.

13. Barger SD, Donoho CJ, Wayment HA. The relative contributions of race/ethnicity, socioeconomic status, health, and social relationships to life satisfaction in the United States. Quality of life research : an international journal of quality of life aspects of treatment, care and rehabilitation. 2009;18(2):179-89.

14. Barghaan D, Harfst T, Watzke B, Dirmaier J, Koch U, Schulz H. Merkmale stationärer psychotherapeutischer Versorgung in Deutschland. PiD - Psychotherapie im Dialog. 2007;8(01):79-84.

15. Barile JP, Mitchell SA, Thompson WW, Zack MM, Reeve BB, Cella D, et al. Patterns of Chronic Conditions and Their Associations With Behaviors and Quality of Life, 2010. Preventing chronic disease. 2015;12:E222.

16. Barkmann C, Otto C, Schon G, Schulte-Markwort M, Schlack R, Ravens-Sieberer U, et al. Modelling trajectories of psychosomatic health complaints in children and adolescents: results of the BELLA study. European child & adolescent psychiatry. 2015;24(6):685-94.

17. Bebbington PE, Meltzer H, Brugha TS, Farrell M, Jenkins R, Ceresa C, et al. Unequal access and unmet need: neurotic disorders and the use of primary care services. Psychological medicine. 2000;30(6):1359-67.

18. Becker N, Abholz HH. Prävalenz und Erkennen von depressiven Störungen in deutschen Allgemeinarztpraxen - eine systematische Literaturübersicht. ZFA (Stuttgart). 2005;81(11):474-81.

19. Behörde für Gesundheit und Verbraucherschutz Hamburg, Behörde für Arbeit S, Familie und Integration Hamburg, . Versorgung psychisch kranker Menschen in Hamburg. 2019.

20. Bennett AC, Gibson C, Rohan AM, Howland JF, Rankin KM. Mental Health and Substance Use-Related Hospitalizations Among Women of Reproductive Age in Illinois and Wisconsin. Public health reports (Washington, DC : 1974). 2019;134(1):17-26.

21. Bergeron L, Valla JP, Breton JJ, Gaudet N, Berthiaume C, Lambert J, et al. Correlates of mental disorders in the Quebec general population of 6 to 14-year olds. Journal of abnormal child psychology. 2000;28(1):47-62.

22. Bermejo I, Klärs G, Böhm K, Hundertmark-Mayser J, Lampert T, Maschewsky-Schneider U, et al. Evaluation des nationalen Gesundheitsziels „Depressive Erkrankungen: verhindern, früh erkennen, nachhaltig behandeln“. Bundesgesundheitsblatt, Gesundheitsforschung, Gesundheitsschutz. 2009;52:897-904.

23. Beutel ME, Klein EM, Brahler E, Reiner I, Junger C, Michal M, et al. Loneliness in the general population: prevalence, determinants and relations to mental health. BMC psychiatry. 2017;17(1):97.

24. Beydoun HA, Williams M, Beydoun MA, Eid SM, Zonderman AB. Relationship of Physical Intimate Partner Violence with Mental Health Diagnoses in the Nationwide Emergency Department Sample. Journal of women's health (2002). 2017;26(2):141-51.

25. Beyers JM, Bates JE, Pettit GS, Dodge KA. Neighborhood structure, parenting processes, and the development of youths' externalizing behaviors: a multilevel analysis. American journal of community psychology. 2003;31(1-2):35-53.

26. Black DR, Held ML. Cardiovascular risk screening for individuals with serious mental illness. Social work in health care. 2017;56(9):809-21.

27. Blais C, Jean S, Sirois C, Rochette L, Plante C, Larocque I, et al. Quebec Integrated Chronic Disease Surveillance System (QICDSS), an innovative approach. Chronic diseases and injuries in Canada. 2014;34(4):226-35.

28. BPtK. BPtK-Studie zur Arbeits- und Erwerbsunfähigkeit. Psychische Erkrankungen und gesundheitsbedingte Frühverrentungen. 2013.

29. BPtK. BPtK-Studie zur Arbeitsunfähigkeit. Psychische Erkrankungen und Krankengeldmanagement. 2015.

30. BPtK. Die Qualität der Versorgung in Psychiatrie und Psychosomatik. Eine Auswertung der Qualitätsberichte der Krankenhäuser. 2016.

31. BPtK. Ein Jahr nach der Reform der Psychotherapie-Richtlinie. Wartezeiten 2018. 2018.

32. Bracke PF, Colman E, Symoens SA, Van Praag L. Divorce, divorce rates, and professional care seeking for mental health problems in Europe: a cross-sectional population-based study. BMC public health. 2010;10:224.

33. Bramesfeld A, Riedel-Heller S. [Priorities in mental health services research]. Psychiatr Prax. 2008;35(7):315-7.

34. Brandstetter S, Dodoo-Schittko F, Speerforck S, Apfelbacher C, Grabe H-J, Jacobi F, et al. Trends in non-help-seeking for mental disorders in Germany between 1997-1999 and 2009-2012: a repeated cross-sectional study. Social psychiatry and psychiatric epidemiology. 2017:1005-13.

35. Breslau J, Miller E, Jin R, Sampson NA, Alonso J, Andrade LH, et al. A multinational study of mental disorders, marriage, and divorce. Acta psychiatrica Scandinavica. 2011;124(6):474-86.

36. Bright MA, Knapp C, Hinojosa MS, Alford S, Bonner B. The Comorbidity of Physical, Mental, and Developmental Conditions Associated with Childhood Adversity: A Population Based Study. Maternal and child health journal. 2016;20(4):843-53.

37. Broussard DL, Sappenfield WB, Fussman C, Kroelinger CD, Grigorescu V. Core state preconception health indicators: a voluntary, multi-state selection process. Maternal and child health journal. 2011;15(2):158-68.

38. Brown DS, Thompson WW, Zack MM, Arnold SE, Barile JP. Associations between health-related quality of life and mortality in older adults. Prevention science : the official journal of the Society for Prevention Research. 2015;16(1):21-30.

39. Brown DW, Balluz LS, Ford ES, Giles WH, Strine TW, Moriarty DG, et al. Associations between short- and long-term unemployment and frequent mental distress among a national sample of men and women. Journal of occupational and environmental medicine. 2003;45(11):1159-66.

40. Brüggemann S, Nebe A, Rose A, Widera T, Buschmann-Steinhage R, Weinbrenner S. Bedeutung psychischer Erkrankungen in der Rehabilitation und Erwerbsminderung. Gesundheitswesen (Bundesverband der Arzte des Offentlichen Gesundheitsdienstes (Germany)). 2014;76(08/09):A19.

41. Bruning J, Arif AA, Rohrer JE. Medical cost and frequent mental distress among the non-elderly US adult population. Journal of public health (Oxford, England). 2014;36(1):134-9.

42. Bundesregierung. Arbeitsbedingungen und psychische Belastungen. Deutscher Bundestag; 2016.

43. Bundesregierung. Sektorenübergreifende Versorgung von Menschen mit psychischen Erkrankungen Deutscher Bundestag; 2019.

44. Bundesregierung. Arbeitsbezogene psychische Belastungen in Deutschland. Deutscher Bundestag; 2019.

45. Bundesregierung. Psychotherapeutische Versorgung von Menschen mit psychischen Erkrankungen. Deutscher Bundestag; 2019.

46. Bunting B, Murphy S, O'Neill S, Ferry F. Prevalence and treatment of 12-month DSM-IV disorders in the Northern Ireland study of health and stress. Social psychiatry and psychiatric epidemiology. 2013;48(1):81-93.

47. Burger H, Boks MP, Hartman CA, Aukes MF, Verhulst FC, Ormel J, et al. Risk score for predicting adolescent mental health problems among children using parental report only: the TRAILS study. Academic pediatrics. 2014;14(6):589-96.

48. Burla L, Diebold M, Dutoit L, Hedinger D, Kohler D, Mercay C, et al. Indikatoren «Gesundheit 2020» Aktualisierung 2017. Neuchâtel2017.

49. Calmeiro L, Camacho I, de Matos MG. Life Satisfaction in Adolescents: The Role of Individual and Social Health Assets. The Spanish journal of psychology. 2018;21:E23.

50. Carder M, Turner S, McNamee R, Agius R. Work-related mental ill-health and 'stress' in the UK (2002-05). Occupational medicine (Oxford, England). 2009;59(8):539-44.

51. Cawthorpe D. A novel population-based health index for mental disorder. The Permanente journal. 2013;17(2):50-4.

52. Centers for Disease Control and Prevention, editor. Mental Illness Surveillance Among Adults in the United States. Atlanta2011.

53. Centers for Disease Control and Prevention. Chronic Disease Indicators - Indicator Definitions - Mental Health Atlanta2011 [Available from: <https://www.cdc.gov/cdi/definitions/mental-health.html>.

54. Chan Chee C, Gourier-Frery C, Guignard R, Beck F. [The current state of mental health surveillance in France]. Sante publique (Vandoeuvre-les-Nancy, France). 2011;23 Suppl 6:S13-29.

55. Chaney EH, Chaney JD, Wang MQ, Eddy JM. Lifestyle behaviors and mental health of American adults. Psychological reports. 2007;100(1):294-302.

56. Cherry NM, Chen Y, McDonald JC. Reported incidence and precipitating factors of work-related stress and mental ill-health in the United Kingdom (1996-2001). Occupational medicine (Oxford, England). 2006;56(6):414-21.

57. Chowdhury PP, Balluz L, Strine TW. Health-related quality of life among minority populations in the United States, BRFSS 2001-2002. Ethnicity & disease. 2008;18(4):483-7.

58. Claassen D, Priebe S. Deutschsprachige psychiatrische Versorgungsforschung - Was und wie wird berichtet? Psychiat Prax. 2003;30(08):414-23.

59. Clark DM, Canvin L, Green J, Layard R, Pilling S, Janecka M. Transparency about the outcomes of mental health services (IAPT approach): an analysis of public data. Lancet (London, England). 2018;391(10121):679-86.

60. Coelho FM, Pinheiro RT, Horta BL, Magalhaes PV, Garcias CM, Silva CV. Common mental disorders and chronic non-communicable diseases in adults: a population-based study. Cadernos de saude publica. 2009;25(1):59-67.

61. Coid J. Epidemiology, public health and the problem of personality disorder. The British journal of psychiatry Supplement. 2003;44:S3-10.

62. Collaborators GEMRMH. The burden of mental disorders in the Eastern Mediterranean region, 1990-2015: findings from the global burden of disease 2015 study. International journal of public health. 2018;63(Suppl 1):25-37.

63. Collishaw S, Maughan B, Goodman R, Pickles A. Time trends in adolescent mental health. Journal of child psychology and psychiatry, and allied disciplines. 2004;45(8):1350-62.

64. Colpe LJ, Barker PR, Karg RS, Batts KR, Morton KB, Gfroerer JC, et al. The National Survey on Drug Use and Health Mental Health Surveillance Study: calibration study design and field procedures. International journal of methods in psychiatric research. 2010;19 Suppl 1:36-48.

65. Conway KP, Green VR, Kasza KA, Silveira ML, Borek N, Kimmel HL, et al. Co-occurrence of tobacco product use, substance use, and mental health problems among adults: Findings from Wave 1 (2013-2014) of the Population Assessment of Tobacco and Health (PATH) Study. Drug and alcohol dependence. 2017;177:104-11.

66. Cook JA, Razzano LA, Swarbrick MA, Jonikas JA, Yost C, Burke L, et al. Health risks and changes in self-efficacy following community health screening of adults with serious mental illnesses. PloS one. 2015;10(4):e0123552.

67. Corscadden L, Callander EJ, Topp SM. International comparisons of disparities in access to care for people with mental health conditions. The International journal of health planning and management. 2018;33(4):967-95.

68. Dadds MR, Collins DAJ, Doyle FL, Tully LA, Hawes DJ, Lenroot RK, et al. A benchmarking study of father involvement in Australian child mental health services. PloS one. 2018;13(8):e0203113.

69. Dal Grande E, Chittleborough CR, Wu J, Shi Z, Goldney RD, Taylor AW. Effect of social mobility in family financial situation and housing tenure on mental health conditions among South Australian adults: results from a population health surveillance system, 2009 to 2011. BMC public health. 2015;15:675.

70. Dalgard OS, Mykletun A, Rognerud M, Johansen R, Zahl PH. Education, sense of mastery and mental health: results from a nation wide health monitoring study in Norway. BMC psychiatry. 2007;7:20.

71. De Graaf R, Bijl RV, Ravelli A, Smit F, Vollebergh WA. Predictors of first incidence of DSM-III-R psychiatric disorders in the general population: findings from the Netherlands Mental Health Survey and Incidence Study. Acta psychiatrica Scandinavica. 2002;106(4):303-13.

72. DeGuzman PB, Merwin EI, Bourguignon C. Population density, distance to public transportation, and health of women in low-income neighborhoods. Public health nursing (Boston, Mass). 2013;30(6):478-90.

73. Deutsche Rentenversicherung. Positionspapier der Deutschen Rentenversicherung zur Bedeutung psychischer Erkrankungen in der Rehabilitation und bei Erwerbsminderung. 2014.

74. Dey M, Mohler-Kuo M, Landolt MA. Health-related quality of life among children with mental health problems: a population-based approach. Health and quality of life outcomes. 2012;10:73.

75. DGPPN. Zahlen und Fakten der Psychiatrie und Psychotherapie. 2019.

76. Dhingra SS, Strine TW, Holt JB, Berry JT, Mokdad AH. Rural-urban variations in psychological distress: findings from the Behavioral Risk Factor Surveillance System, 2007. International journal of public health. 2009;54 Suppl 1:16-22.

77. Dhingra SS, Zack MM, Strine TW, Druss BG, Berry JT, Balluz LS. Psychological distress severity of adults reporting receipt of treatment for mental health problems in the BRFSS. Psychiatric services (Washington, DC). 2011;62(4):396-403.

78. Diaz-Granados N, McDermott S, Wang F, Posada-Villa J, Saavedra J, Rondon MB, et al. Monitoring gender equity in mental health in a low-, middle-, and high-income country in the Americas. Psychiatric services (Washington, DC). 2011;62(5):516-24.

79. Dreßing H, Salize H-J. Zwangsunterbringung und Zwangsbehandlung psychisch Kranker in den Mitgliedsländern der Europäischen Union. Psychiat Prax. 2004;31(01):34-9.

80. Drukker M, Bak M, Campo J, Driessen G, Van Os J, Delespaul P. The cumulative needs for care monitor: a unique monitoring system in the south of the Netherlands. Social psychiatry and psychiatric epidemiology. 2010;45(4):475-85.

81. Druss BG, Zhao L, Von Esenwein S, Morrato EH, Marcus SC. Understanding excess mortality in persons with mental illness: 17-year follow up of a nationally representative US survey. Medical care. 2011;49(6):599-604.

82. Duckers ML, Brewin CR. A Paradox in Individual Versus National Mental Health Vulnerability: Are Higher Resource Levels Associated With Higher Disorder Prevalence? Journal of traumatic stress. 2016;29(6):572-6.

83. Durbin J, Lin E, Layne C, Teed M. Is readmission a valid indicator of the quality of inpatient psychiatric care? The journal of behavioral health services & research. 2007;34(2):137-50.

84. Durbin J, Prendergast P, Dewa CS, Rush B, Cooke RG. Mental health program monitoring: towards simplifying a complex task. Psychiatric rehabilitation journal. 2003;26(3):249-61.

85. Dwyer-Lindgren L, Mackenbach JP, van Lenthe FJ, Mokdad AH. Self-reported general health, physical distress, mental distress, and activity limitation by US county, 1995-2012. Population health metrics. 2017;15:16.

86. Edlund MJ, Wang J, Brown KG, Forman-Hoffman VL, Calvin SL, Hedden SL, et al. Which mental disorders are associated with the greatest impairment in functioning? Social psychiatry and psychiatric epidemiology. 2018;53(11):1265-76.

87. Erol N, Simsek Z, Oner O, Munir K. Behavioral and emotional problems among Turkish children at ages 2 to 3 years. Journal of the American Academy of Child and Adolescent Psychiatry. 2005;44(1):80-7.

88. European Commission. POSTER - The European core Health Indicators (ECHI) shortlist of 88 health indicators identified by policy area 2013 [Available from: <https://ec.europa.eu/health/indicators_data/publications_en>.

89. Farr SL, Bish CL. Preconception health among women with frequent mental distress: a population-based study. Journal of women's health (2002). 2013;22(2):153-8.

90. Farrell M, Howes S, Taylor C, Lewis G, Jenkins R, Bebbington P, et al. Substance misuse and psychiatric comorbidity: an overview of the OPCS National Psychiatric Morbidity Survey. International review of psychiatry (Abingdon, England). 2003;15(1-2):43-9.

91. Ferrer RL, Palmer R. Variations in health status within and between socioeconomic strata. Journal of epidemiology and community health. 2004;58(5):381-7.

92. Fleury MJ, Fortin M, Rochette L, Grenier G, Huynh C, Pelletier E, et al. Assessing quality indicators related to mental health emergency room utilization. BMC emergency medicine. 2019;19(1):8.

93. Fontes LFC, Conceicao OC, Machado S. Childhood and adolescent sexual abuse, victim profile and its impacts on mental health. Ciencia & saude coletiva. 2017;22(9):2919-28.

94. Forman-Hoffman V, Batts K, Bose J, Glasheen C, Hirsch E, Yu F, et al. Correlates of exposure to potentially traumatic experiences: Results from a national household survey. Psychological trauma : theory, research, practice and policy. 2019;11(3):360-7.

95. Forman-Hoffman VL, Batts KR, Hedden SL, Spagnola K, Bose J. Comorbid mental disorders among adults in the mental health surveillance survey. Annals of epidemiology. 2018;28(7):468-74.

96. Forsell Y. Psychiatric symptoms, social disability, low wellbeing and need for treatment: data from a population-based study. The International journal of social psychiatry. 2004;50(3):195-203.

97. Forsell Y. The pathway to meeting need for mental health services in Sweden. Psychiatric services (Washington, DC). 2006;57(1):114-9.

98. Fryers T, Brugha T, Morgan Z, Smith J, Hill T, Carta M, et al. Prevalence of psychiatric disorder in Europe: the potential and reality of meta-analysis. Social psychiatry and psychiatric epidemiology. 2004;39(11):899-905.

99. Fujisawa R. Mental health indicators: within 30-day hospital re-admission. In: OECD, editor. OECD HCQI Expert Meeting2012.

100. Fujishiro K. Is perceived racial privilege associated with health? Findings from the Behavioral Risk Factor Surveillance System. Social science & medicine (1982). 2009;68(5):840-4.

101. Gaebel W, Kowitz S, Fritze J, Zielasek J. Inanspruchnahme des Versorgungssystems bei psychischen Erkrankungen. Dtsch Arztebl International. 2013;110(47):799-808.

102. Gaebel W, Zielasek J, Kowitz S. Nutzung von Routinedaten für die psychiatrische und psychosomatische Versorgungsforschung. Die Psychiatrie. 2011;08(01):23-33.

103. Garcia EL, Banegas JR, Perez-Regadera AG, Cabrera RH, Rodriguez-Artalejo F. Social network and health-related quality of life in older adults: a population-based study in Spain. Quality of life research : an international journal of quality of life aspects of treatment, care and rehabilitation. 2005;14(2):511-20.

104. Garrido-Cumbrera M, Almenara-Barrios J, Lopez-Lara E, Peralta-Saez JL, Garcia-Gutierrez JC, Salvador-Carulla L. Development and spatial representation of synthetic indexes of outpatient mental health care in Andalusia (Spain). Epidemiologia e psichiatria sociale. 2008;17(3):192-200.

105. Gater R, Chisholm D, Dowrick C. Mental health surveillance and information systems. Eastern Mediterranean health journal = La revue de sante de la Mediterranee orientale = al-Majallah al-sihhiyah li-sharq al-mutawassit. 2015;21(7):512-6.

106. Gesundheit Bf. Bericht: 6. nationales Gesundheitsziel – Depressive Erkrankungen: verhindern, früh erkennen, nachhaltig behandeln. . Berlin: BMG; 2006.

107. Glover G, Arts G, Wooff D. A needs index for mental health care in England based on updatable data. Social psychiatry and psychiatric epidemiology. 2004;39(9):730-8.

108. Gómez-Beneyto M, Rodriguez Escobar J, Rasillo Rodríguez E, Gómez González B, Blanco Quintana A, Vannerau Sánchez D, et al. Mental Health Strategy of the Spanish National Health System 2009-2013 Madrid: Ministry of Health, Social Services and Equality; 2012.

109. Gosswald A, Lange M, Kamtsiuris P, Kurth BM. [DEGS: German Health Interview and Examination Survey for Adults. A nationwide cross-sectional and longitudinal study within the framework of health monitoring conducted by the Robert Koch Institute]. Bundesgesundheitsblatt, Gesundheitsforschung, Gesundheitsschutz. 2012;55(6-7):775-80.

110. Government United Kingdom. No health without mental health. Mental health dashboard. Applies to: England. In: Department of Health, editor. 2013.

111. Grabe HJ, Alte D, Adam C, Sauer S, John U, Freyberger HJ. Seelische Belastung und Inanspruchnahme psychiatrischer und psychotherapeutischer Versorgung. Psychiat Prax. 2005;32(06):299-303.

112. Grossimlinghaus I, Falkai P, Gaebel W, Hasan A, Janner M, Janssen B, et al. [Assessment of quality indicators with routine data: Presentation of a feasibility test in ten specialist clinics for psychiatry and psychotherapy]. Der Nervenarzt. 2015;86(11):1393-9.

113. Hacker K, Drainoni ML. Mental health and illness in Boston's children and adolescents: one city's experience and its implications for mental health policy makers. Public health reports (Washington, DC : 1974). 2001;116(4):317-26.

114. Hagerty MR. Social comparisons of income in one's community: evidence from national surveys of income and happiness. Journal of personality and social psychology. 2000;78(4):764-71.

115. Hancock T. Indicators of environmental health in the urban setting. Canadian journal of public health = Revue canadienne de sante publique. 2002;93 Suppl 1:S45-51.

116. Harford TC, Chen CM, Grant BF. DSM-IV Personality Disorders: Dimensional Ordered Categorization and Associations With Disability and Selected Axis I Disorders in a General Population Survey. Journal of personality disorders. 2015;29(5):627-40.

117. Harris RB, Stanley J, Cormack DM. Racism and health in New Zealand: Prevalence over time and associations between recent experience of racism and health and wellbeing measures using national survey data. PloS one. 2018;13(5):e0196476.

118. Hart C, de Vet R, Moran P, Hatch SL, Dean K. A UK population-based study of the relationship between mental disorder and victimisation. Social psychiatry and psychiatric epidemiology. 2012;47(10):1581-90.

119. Healthybelgium. For a healthy Belgium: health and healthcare indicators 2021 [Available from: <https://www.healthybelgium.be/en/>.

120. Henderson S, Andrews G, Hall W. Australia's mental health: an overview of the general population survey. The Australian and New Zealand journal of psychiatry. 2000;34(2):197-205.

121. Hermann R, Mattke S, et al. Selecting indicators for the quality of mental health care at health systems level in OECD countries. OECD Health Technical Papers No. 17. Paris: OECD Publishing; 2004.

122. Hewlett E, Moran V. Making Mental Health Count: The Social and Economic Costs of Neglecting Mental Health Care. Paris: OECD Publishing; 2014.

123. Holger S, Baarghan D, Harfst T, Koch U. Psychotherapeutische Versorgung. Berlin; 2008.

124. Holzhausen M, Fuchs J, Busch M, Ernert A, Six-Merker J, Knopf H, et al. Operationalizing multimorbidity and autonomy for health services research in aging populations--the OMAHA study. BMC health services research. 2011;11:47.

125. Holzinger A, Floris F, Schomerus G, Carta MG, Angermeyer MC. Gender differences in public beliefs and attitudes about mental disorder in western countries: a systematic review of population studies. Epidemiology and psychiatric sciences. 2012;21(1):73-85.

126. Honjo K, Kawakami N, Tsuchiya M, Sakurai K. Association of subjective and objective socioeconomic status with subjective mental health and mental disorders among Japanese men and women. International journal of behavioral medicine. 2014;21(3):421-9.

127. Hopkins RS, Landen M, Toe M. Development of Indicators for Public Health Surveillance of Substance Use and Mental Health. Public health reports (Washington, DC : 1974). 2018;133(5):523-31.

128. Hübner-Liebermann B, Hajak G, Spießl H. Versorgungsepidemiologie: Entwicklung in der stationär-psychiatrischen Versorgung 1996–2006. Psychiat Prax. 2008;35(08):387-91.

129. Hudson CG, Abbott MW. Modeling the geographic distribution of serious mental illness in New Zealand. Social psychiatry and psychiatric epidemiology. 2013;48(1):25-36.

130. Huynh Q, Craig W, Janssen I, Pickett W. Exposure to public natural space as a protective factor for emotional well-being among young people in Canada. BMC public health. 2013;13:407.

131. IGES. Strukturen und Finanzierung der neurologischen und psychiatrischen Versorgung. 2007.

132. Iwasa H, Masui Y, Gondo Y, Inagaki H, Kawaai C, Suzuki T. Personality and all-cause mortality among older adults dwelling in a Japanese community: a five-year population-based prospective cohort study. The American journal of geriatric psychiatry : official journal of the American Association for Geriatric Psychiatry. 2008;16(5):399-405.

133. Jacob KS, Sharan P, Mirza I, Garrido-Cumbrera M, Seedat S, Mari JJ, et al. Mental health systems in countries: where are we now? Lancet (London, England). 2007;370(9592):1061-77.

134. Jacobi F, Mack S, Gerschler A, Scholl L, Hofler M, Siegert J, et al. The design and methods of the mental health module in the German Health Interview and Examination Survey for Adults (DEGS1-MH). International journal of methods in psychiatric research. 2013;22(2):83-99.

135. Jacobi F, Wittchen HU, Holting C, Hofler M, Pfister H, Muller N, et al. Prevalence, co-morbidity and correlates of mental disorders in the general population: results from the German Health Interview and Examination Survey (GHS). Psychological medicine. 2004;34(4):597-611.

136. Jacobi F, Wittchen HU, Holting C, Sommer S, Lieb R, Hofler M, et al. Estimating the prevalence of mental and somatic disorders in the community: aims and methods of the German National Health Interview and Examination Survey. International journal of methods in psychiatric research. 2002;11(1):1-18.

137. Jager M, Rossler W. Epidemiology of mental health care. Der Nervenarzt. 2012;83(3):389-401; quiz 2.

138. Jager M, Sobocki P, Rossler W. Cost of disorders of the brain in Switzerland with a focus on mental disorders. Swiss medical weekly. 2008;138(1-2):4-11.

139. Janssen EM, McGinty EE, Azrin ST, Juliano-Bult D, Daumit GL. Review of the evidence: prevalence of medical conditions in the United States population with serious mental illness. General hospital psychiatry. 2015;37(3):199-222.

140. Janssen MM, Wensing M, van der Gaag RJ, van Deurzen PA, Buitelaar JK. [Adjustment and alteration of the quality indicators in the Basic Set (GGZ) for use in child and adolescent psychiatry]. Tijdschrift voor psychiatrie. 2013;55(1):21-31.

141. Janssen WA, van de Sande R, Noorthoorn EO, Nijman HL, Bowers L, Mulder CL, et al. Methodological issues in monitoring the use of coercive measures. International journal of law and psychiatry. 2011;34(6):429-38.

142. Jenkins R, Bhugra D, Bebbington P, Brugha T, Farrell M, Coid J, et al. Debt, income and mental disorder in the general population. Psychological medicine. 2008;38(10):1485-93.

143. Jia H, Lubetkin EI. Time trends and seasonal patterns of health-related quality of life among U.S. adults. Public health reports (Washington, DC : 1974). 2009;124(5):692-701.

144. Jia H, Moriarty DG, Kanarek N. County-level social environment determinants of health-related quality of life among US adults: a multilevel analysis. Journal of community health. 2009;34(5):430-9.

145. John U, Meyer C, Rumpf HJ, Schumann A, Dilling H, Hapke U. Self-rated general health and psychiatric disorders in a general population sample. European psychiatry : the journal of the Association of European Psychiatrists. 2005;20(3):223-8.

146. Jordans MJ, Chisholm D, Semrau M, Upadhaya N, Abdulmalik J, Ahuja S, et al. Indicators for routine monitoring of effective mental healthcare coverage in low- and middle-income settings: a Delphi study. Health policy and planning. 2016;31(8):1100-6.

147. Jorm AF, Butterworth P. Changes in psychological distress in Australia over an 8-year period: evidence for worsening in young men. The Australian and New Zealand journal of psychiatry. 2006;40(1):47-50.

148. Jorm AF, Reavley NJ. Changes in psychological distress in Australian adults between 1995 and 2011. The Australian and New Zealand journal of psychiatry. 2012;46(4):352-6.

149. Joska J, Flisher AJ. The assessment of need for mental health services. Social psychiatry and psychiatric epidemiology. 2005;40(7):529-39.

150. Kahumoku EP, Vazsonyi AT, Pagava K, Phagava H, Alsaker FD, Michaud PA. Objectified body consciousness and mental health in female adolescents: cross-cultural evidence from Georgian and Swiss national samples. The Journal of adolescent health : official publication of the Society for Adolescent Medicine. 2011;49(2):141-7.

151. Kaneko Y, Motohashi Y. Male gender and low education with poor mental health literacy: a population-based study. Journal of epidemiology. 2007;17(4):114-9.

152. Kapi A, Veltsista A, Kavadias G, Lekea V, Bakoula C. Social determinants of self-reported emotional and behavioral problems in Greek adolescents. Social psychiatry and psychiatric epidemiology. 2007;42(7):594-8.

153. Karunanayake CP, Pahwa P. Statistical modelling of mental distress among rural and urban seniors. Chronic diseases in Canada. 2009;29(3):118-27.

154. Kassenärztliche Bundesvereinigung (KBV). Statistische Informationen aus dem Bundesarztregister. <http://www.kbv.de/media/sp/2017_12_31_BAR_Statistik.pdf>. Zugegriffen: 19. Juli 2018. 2017.

155. Katschnig H. Monitoring service utilization of persons with mental disorders--a case for mapping pathways of care. Epidemiology and psychiatric sciences. 2011;20(1):7-13.

156. Kendall T, Glover N, Taylor C, Pilling S. Quality, bias and service user experience in healthcare: 10 years of mental health guidelines at the UK National Collaborating Centre for Mental Health. International review of psychiatry (Abingdon, England). 2011;23(4):342-51.

157. Kenning C, Coventry PA, Gibbons C, Bee P, Fisher L, Bower P. Does patient experience of multimorbidity predict self-management and health outcomes in a prospective study in primary care? Family practice. 2015;32(3):311-6.

158. Kisely S, Lesage A. [Mental health services in Australia]. Sante mentale au Quebec. 2014;39(1):195-208.

159. Kisely S, Lin E, Lesage A, Gilbert C, Smith M, Campbell LA, et al. Use of administrative data for the surveillance of mental disorders in 5 provinces. Canadian journal of psychiatry Revue canadienne de psychiatrie. 2009;54(8):571-5.

160. Kisely S, Smith M, Lawrence D, Maaten S. Mortality in individuals who have had psychiatric treatment: population-based study in Nova Scotia. The British journal of psychiatry : the journal of mental science. 2005;187:552-8.

161. Kloos B, Shah S. A social ecological approach to investigating relationships between housing and adaptive functioning for persons with serious mental illness. American journal of community psychology. 2009;44(3-4):316-26.

162. Kobau R, Safran MA, Zack MM, Moriarty DG, Chapman D. Sad, blue, or depressed days, health behaviors and health-related quality of life, Behavioral Risk Factor Surveillance System, 1995-2000. Health and quality of life outcomes. 2004;2:40.

163. Kohler L. Monitoring children's health and well-being by indicators and index: apples and oranges or fruit salad? Child: care, health and development. 2016;42(6):798-808.

164. Kontopantelis E, Olier I, Planner C, Reeves D, Ashcroft DM, Gask L, et al. Primary care consultation rates among people with and without severe mental illness: a UK cohort study using the Clinical Practice Research Datalink. BMJ open. 2015;5(12):e008650.

165. Korkeila J, Lehtinen V, Bijl R, Odd-Steffer D, Kovess V, Morgan A, et al. Establishing a set of mental health indicators for Europe. Scandinavian journal of public health. 2003;31:451-9.

166. Korkeila JA, Tuohimaki C, Kaltiala-Heino R, Lehtinen V, Joukamaa M. Predicting use of coercive measures in Finland. Nordic journal of psychiatry. 2002;56(5):339-45.

167. Kowitz S, Zielasek J, Gaebel W. Die Versorgungssituation bei psychischen Störungen in Deutschland. Dtsch med Wochenschr. 2014;139(23):1249-52.

168. Kratochwill TR, Albers CA, Shernoff ES. School-based interventions. Child and adolescent psychiatric clinics of North America. 2004;13(4):885-903, vi-vii.

169. Kristensen S, Mainz J, Baandrup L, Bonde M, Videbech P, Holmskov J, et al. Conceptualizing patient-reported outcome measures for use within two Danish psychiatric clinical registries: description of an iterative co-creation process between patients and healthcare professionals. Nordic journal of psychiatry. 2018;72(6):409-19.

170. Kroenke K, Unutzer J. Closing the False Divide: Sustainable Approaches to Integrating Mental Health Services into Primary Care. Journal of general internal medicine. 2017;32(4):404-10.

171. Kroese FM, Evers C, Adriaanse MA, de Ridder DTD. Bedtime procrastination: A self-regulation perspective on sleep insufficiency in the general population. Journal of health psychology. 2016;21(5):853-62.

172. Krueger RF, Caspi A, Moffitt TE. Epidemiological personology: the unifying role of personality in population-based research on problem behaviors. Journal of personality. 2000;68(6):967-98.

173. Kruse J, Herzog W. Zwischenbericht zum Gutachten "Zur ambulanten psychosomatischen/psychotherapeutischen Versorgung in der kassenärztlichen Versorgung in Deutschland - Formen der Versorgung und ihre Effizienz" im Auftrag der Kassenärztlichen Bundesvereinigung (KBV). 2012.

174. Kunze B, Wang B, Isensee C, Schlack R, Ravens-Sieberer U, Klasen F, et al. Gender associated developmental trajectories of SDQ-dysregulation profile and its predictors in children. Psychological medicine. 2018;48(3):404-15.

175. Kuosmanen L, Kaltiala-Heino R, Suominen S, Karkkainen J, Hatonen H, Ranta S, et al. Patient complaints in Finland 2000-2004: a retrospective register study. Journal of medical ethics. 2008;34(11):788-92.

176. Kurdyak P, Wiesenfeld L, Sockalingam S. Choosing Wisely? Let's Start with Working Wisely. Canadian journal of psychiatry Revue canadienne de psychiatrie. 2016;61(1):25-8.

177. Kwan B, Rickwood DJ. A systematic review of mental health outcome measures for young people aged 12 to 25 years. BMC psychiatry. 2015;15:279.

178. Lamont A, Ukoumunne OC, Tyrer P, Thornicroft G, Patel R, Slaughter J. The geographical mobility of severely mentally ill residents in London. Social psychiatry and psychiatric epidemiology. 2000;35(4):164-9.

179. Lange C, Jentsch F, Allen J, Hoebel J, Kratz AL, von der Lippe E, et al. Data Resource Profile: German Health Update (GEDA)--the health interview survey for adults in Germany. International journal of epidemiology. 2015;44(2):442-50.

180. Lauber C, Nordt C, Rossler W. Recommendations of mental health professionals and the general population on how to treat mental disorders. Social psychiatry and psychiatric epidemiology. 2005;40(10):835-43.

181. Lauer EA, Lauer E. Assessing the association between mental health and disability indicators among adults living in the United States. Disability and health journal. 2019;12(1):98-105.

182. Lawrence RJ. Urban environmental health indicators: appraisal and policy directives. Reviews on environmental health. 2008;23(4):299-325.

183. Leao LH, Gomez CM. The issue of mental health in occupational health surveillance. Ciencia & saude coletiva. 2014;19(12):4649-58.

184. Lechner S, Herzog W, Boehlen F, Maatouk I, Saum KU, Brenner H, et al. Control preferences in treatment decisions among older adults - Results of a large population-based study. Journal of psychosomatic research. 2016;86:28-33.

185. Lehmann I, Chisholm D, Hinkov H, Hoschl C, Kapocs G, Kurimay T, et al. Development of quality indicators for mental healthcare in the Danube region. Psychiatria Danubina. 2018;30(2):197-206.

186. Lehtinen V. Building up good mental health. Guidelines based on existing knowledge. Helsinki: MMHE Project; 2008.

187. Lepine JP, Gasquet I, Kovess V, Arbabzadeh-Bouchez S, Negre-Pages L, Nachbaur G, et al. [Prevalence and comorbidity of psychiatric disorders in the French general population]. L'Encephale. 2005;31(2):182-94.

188. Lercher P, Evans GW, Meis M, Kofler WW. Ambient neighbourhood noise and children's mental health. Occupational and environmental medicine. 2002;59(6):380-6.

189. Lesage A, Rochette L, Emond V, Pelletier E, St-Laurent D, Diallo FB, et al. A Surveillance System to Monitor Excess Mortality of People With Mental Illness in Canada. Canadian journal of psychiatry Revue canadienne de psychiatrie. 2015;60(12):571-9.

190. Levin KA, Currie C, Muldoon J. Mental well-being and subjective health of 11- to 15-year-old boys and girls in Scotland, 1994-2006. European journal of public health. 2009;19(6):605-10.

191. Lim KL, Jacobs P, Ohinmaa A, Schopflocher D, Dewa CS. A new population-based measure of the economic burden of mental illness in Canada. Chronic diseases in Canada. 2008;28(3):92-8.

192. Lindesay J, Baillon S, Brugha T, Dennis M, Stewart R, Araya R, et al. Worry content across the lifespan: an analysis of 16- to 74-year-old participants in the British National Survey of Psychiatric Morbidity 2000. Psychological medicine. 2006;36(11):1625-33.

193. Lindstrom M, Rosvall M. Parental separation in childhood and self-reported psychological health: A population-based study. Psychiatry research. 2016;246:783-8.

194. Lloyd-Evans B, Paterson B, Onyett S, Brown E, Istead H, Gray R, et al. National implementation of a mental health service model: A survey of Crisis Resolution Teams in England. International journal of mental health nursing. 2018;27(1):214-26.

195. Lohmann-Haislah A. Stressreport Deutschland 2012. Psychische Anforderungen, Ressourcen und Befinden>. In: BAuA, editor. Dortmund/Berlin/Dresden2012.

196. Lora A, Bezzi R, Di Vietri R, Gandini A, Spinogatti F, Zocchetti C. [Packages of care in the departments of mental health in Lombardy]. Epidemiologia e psichiatria sociale. 2002;11(2):100-15.

197. Mack S, Jacobi F, Gerschler A, Strehle J, Höfler M, Busch MA, et al. Self-reported utilization of mental health services in the adult German population - evidence for unmet needs? Results of the DEGS1-Mental Health Module (DEGS1-MH). International journal of methods in psychiatric research. 2014;23:289-303.

198. Magee T, Sister Callista R. Predicting school-age behavior problems: the role of early childhood risk factors. Pediatric nursing. 2008;34(1):37-44.

199. Marryat L, Thompson L, Minnis H, Wilson P. Primary schools and the amplification of social differences in child mental health: a population-based cohort study. Journal of epidemiology and community health. 2018;72(1):27-33.

200. Martin CM. What matters in "multimorbidity"? Arguably resilience and personal health experience are central to quality of life and optimizing survival. Journal of evaluation in clinical practice. 2018;24(6):1282-4.

201. Mathiesen KS, Sanson A. Dimensions of early childhood behavior problems: stability and predictors of change from 18 to 30 months. Journal of abnormal child psychology. 2000;28(1):15-31.

202. McChesney GC, Adamson G, Shevlin M. A latent class analysis of trauma based on a nationally representative sample of US adolescents. Social psychiatry and psychiatric epidemiology. 2015;50(8):1207-17.

203. McKenzie SK, Carter K. Does transition into parenthood lead to changes in mental health? Findings from three waves of a population based panel study. Journal of epidemiology and community health. 2013;67(4):339-45.

204. McKenzie SK, Imlach Gunasekara F, Richardson K, Carter K. Do changes in socioeconomic factors lead to changes in mental health? Findings from three waves of a population based panel study. Journal of epidemiology and community health. 2014;68(3):253-60.

205. Meadows G, Liaw T, Burgess P, Bobevski I, Fossey E. Australian general practice and the meeting of needs for mental health care. Social psychiatry and psychiatric epidemiology. 2001;36(12):595-603.

206. Meijwaard SC, Kikkert M, de Mooij LD, Lommerse NM, Peen J, Schoevers RA, et al. Risk of Criminal Victimisation in Outpatients with Common Mental Health Disorders. PloS one. 2015;10(7):e0128508.

207. Melchior H, Schulz H, Härter M. Faktencheck Gesundheit - Regionale Unterschiede in der Diagnostik und Behandlung von Depressionen. Gütersloh: Bertelsmann Stiftung; 2014 2014.

208. Meldrum ML, Kelly EL, Calderon R, Brekke JS, Braslow JT. Implementation Status of Assisted Outpatient Treatment Programs: A National Survey. Psychiatric services (Washington, DC). 2016;67(6):630-5.

209. Meng X, D'Arcy C. The projected effect of increasing physical activity on reducing the prevalence of common mental disorders among Canadian men and women: a national population-based community study. Preventive medicine. 2013;56(1):59-63.

210. Merchant JA, Kelly KM, Burmeister LF, Lozier MJ, Amendola A, Lind DP, et al. Employment status matters: a statewide survey of quality-of-life, prevention behaviors, and absenteeism and presenteeism. Journal of occupational and environmental medicine. 2014;56(7):686-98.

211. Messias E, Eaton W, Nestadt G, Bienvenu OJ, Samuels J. Psychiatrists' ascertained treatment needs for mental disorders in a population-based sample. Psychiatric services (Washington, DC). 2007;58(3):373-7.

212. Meyer C, Rumpf HJ, Hapke U, Dilling H, John U. [Lifetime prevalence of mental disorders in general adult population. Results of TACOS study]. Der Nervenarzt. 2000;71(7):535-42.

213. Meyer C, Rumpf HJ, Hapke U, John U. Impact of psychiatric disorders in the general population: satisfaction with life and the influence of comorbidity and disorder duration. Social psychiatry and psychiatric epidemiology. 2004;39(6):435-41.

214. Miech R, Power C, Eaton WW. Disparities in psychological distress across education and sex: a longitudinal analysis of their persistence within a cohort over 19 years. Annals of epidemiology. 2007;17(4):289-95.

215. Milder IE, de Hollander EL, Picavet HS, Verschuren WM, de Groot LC, Bemelmans WJ. Changes in weight and health-related quality of life. The Doetinchem Cohort Study. Journal of epidemiology and community health. 2014;68(5):471-7.

216. Ministerium für Arbeit SuIdLS-A. Abschlussbericht. Bestandsaufnahme, Prognose und Handlungsempfehlungen zur Versorgung von psychisch kranken und seelisch behinderten Menschen im Land Sachsen-Anhalt unter Berücksichtigung demographischer und regionaler Entwicklungen. 2018.

217. Ministry of Health, editor. Office of the Director of Mental Health and Addiction Services: Annual Report 2017. Wellington: Ministry of Health; 2019.

218. Mock SE, Eibach RP. Aging attitudes moderate the effect of subjective age on psychological well-being: evidence from a 10-year longitudinal study. Psychology and aging. 2011;26(4):979-86.

219. Moreau-Gruet F. Monitoring zur psychischen Gesundheit – mit Fokus ‹Ältere Menschen› und ‹Stress am Arbeitsplatz›. Aktualisierung 2014. Gesundheitsförderung Schweiz Arbeitspapier 2. Bern und Lausanne: obsan; 2014.

220. Moriarty DG, Kobau R, Zack MM, Zahran HS. Tracking Healthy Days -- a window on the health of older adults. Preventing chronic disease. 2005;2(3):A16.

221. Moriarty DG, Zack MM, Kobau R. The Centers for Disease Control and Prevention's Healthy Days Measures - population tracking of perceived physical and mental health over time. Health and quality of life outcomes. 2003;1:37.

222. Muhajarine N, Labonte R, Winquist BD. The Canadian Index of Wellbeing: key findings from the healthy populations domain. Canadian journal of public health = Revue canadienne de sante publique. 2012;103(5):e342-7.

223. Mulder CL, van der Gaag M, Bruggeman R, Cahn W, Delespaul PAE, Dries P, et al. [Routine Outcome Monitoring for patients with severe mental illness: a consensus document]. Tijdschrift voor psychiatrie. 2010;52(3):169-79.

224. National Institute of Mental Health. Mental Health Information. Statistics Rockwill2021 [Available from: <https://www.nimh.nih.gov/health/statistics/index.shtml>.

225. National Mental Health Commission, editor. Final Report: Expert Reference Group to COAG Working Group on Mental Health Reform on National Targets and Indicators for Mental Health Reform. Sydney, Australia2013.

226. National Mental Health Commission, editor. Mental Health and Suicide Prevention Monitoring and Reporting Framework. Complete Final Report to the National Mental Health Commission with Appendices. Sydney, Australia2018.

227. National Mental Health Performance Subcommittee, editor. The Fourth National Mental Health Plan Measurement Strategy. Canberra: Commonwealth of Australia; 2011.

228. National Research and Development Centre for Welfare and Health. Establishment of a set of mental health indicators for European Union. Helsinki: STAKES; 2001.

229. Ngamini Ngui A, Apparicio P. [Potential accessibility to mental health services in Montreal: a geographical information system approach]. Revue d'epidemiologie et de sante publique. 2011;59(6):369-78.

230. NHS England. Mental Health Five Year Forward View Dashboard. Period: Quarter 4 2018/19. 2019.

231. NHS Health Scotland. National Adult Mental Health and Well-being Indicators for Scotland. Final Briefing December 2007: NHS Health Scotland. Public Health Observatory Division; 2007 [updated 22.11.2018. Available from: <http://www.healthscotland.scot/media/2235/summary-briefing-paper-adult-mental-health-indicators-2007.pdf>.

232. Niclasen B, Kohler L. National indicators of child health and well-being in Greenland. Scandinavian journal of public health. 2009;37(4):347-56.

233. Norlund S, Reuterwall C, Hoog J, Janlert U, Slunga Jarvholm L. Work situation and self-perceived economic situation as predictors of change in burnout--a prospective general population-based cohort study. BMC public health. 2015;15:329.

234. Norwegian Institute of Public Health. Mental illness among adults Oslo2016 [Available from: <https://www.fhi.no/en/op/hin/mental-health/psykisk-helse-hos-voksne/#prevalence-of-mental-disorders-among-adults>.

235. Norwegian Institute of Public Health. Public Health Report Oslo2016 [Available from: <https://www.fhi.no/en/op/hin/>.

236. Nübel J, Müllender S, Hapke U, Jacobi F. Epidemie der Depression? Prävalenzentwicklung und Inanspruchnahme von Hilfs- und Versorgungsangeboten. Der Nervenarzt. 2019.

237. Nübling R. Psychotherapeutische Versorgungsforschung - Status Quo und Perspektiven. Psychotherapieforum Heidelberg2015.

238. Nutsford D, Pearson AL, Kingham S. An ecological study investigating the association between access to urban green space and mental health. Public health. 2013;127(11):1005-11.

239. OECD, editor. Definitions for Health Care Quality Indicators 2016-2017 HCQI Data Collection: OECD; 2016.

240. OECD, editor. Health at a Glance 2017: OECD Indicators. Paris: OECD Publishing; 2017.

241. Office of Disease Prevention and Promotion. Healthy People 2020 Washington: [www.healthypeople.gov](http://www.healthypeople.gov); 2019 [updated 10/08/20. Available from: <https://www.healthypeople.gov/2020/topics-objectives/topic/health-related-quality-of-life-well-being/objectives>.

242. Office of Disease Prevention and Promotion. Healthy People 2020. Mental Health and Mental Disorders Washington: [www.healthypeople.gov](http://www.healthypeople.gov); 2019 [updated 10/08/20. Available from: <https://www.healthypeople.gov/2020/topics-objectives/topic/mental-health-and-mental-disorders>.

243. Office of Disease Prevention and Promotion. Healthy People 2020. Health-Related Quality of Life & Well-Being Washington: [www.healthypeople.gov](http://www.healthypeople.gov); 2019 [updated 10/08/20. Available from: <https://www.healthypeople.gov/2020/topics-objectives/topic/health-related-quality-of-life-well-being/objectives>.

244. Olstad R, Sexton H, Sogaard AJ. The Finnmark Study. A prospective population study of the social support buffer hypothesis, specific stressors and mental distress. Social psychiatry and psychiatric epidemiology. 2001;36(12):582-9.

245. Ormel J, Oldehinkel AJ, Ferdinand RF, Hartman CA, De Winter AF, Veenstra R, et al. Internalizing and externalizing problems in adolescence: general and dimension-specific effects of familial loadings and preadolescent temperament traits. Psychological medicine. 2005;35(12):1825-35.

246. Orpana H, Vachon J, Dykxhoorn J, McRae L, Jayaraman G. Monitoring positive mental health and its determinants in Canada: the development of the Positive Mental Health Surveillance Indicator Framework. Health promotion and chronic disease prevention in Canada: research, policy and practice. 2016;36(1):1-10.

247. Oswald AJ, Wu S. Objective confirmation of subjective measures of human well-being: evidence from the U.S.A. Science (New York, NY). 2010;327(5965):576-9.

248. Packness A, Waldorff FB, Christensen RD, Hastrup LH, Simonsen E, Vestergaard M, et al. Impact of socioeconomic position and distance on mental health care utilization: a nationwide Danish follow-up study. Social psychiatry and psychiatric epidemiology. 2017;52(11):1405-13.

249. Paksarian D, Cui L, Angst J, Ajdacic-Gross V, Rossler W, Merikangas KR. Latent Trajectories of Common Mental Health Disorder Risk Across 3 Decades of Adulthood in a Population-Based Cohort. JAMA psychiatry. 2016;73(10):1023-31.

250. Palin JL, Goldner EM, Koehoorn M, Hertzman C. Prevalence and frequency of mental health care provided by general practitioners: differences between 2 national data sources for the same population. Canadian journal of psychiatry Revue canadienne de psychiatrie. 2012;57(6):366-74.

251. Paris J. Estimating the prevalence of personality disorders in the community. Journal of personality disorders. 2010;24(4):405-11.

252. Park MJ, Paul Mulye T, Adams SH, Brindis CD, Irwin CE, Jr. The health status of young adults in the United States. The Journal of adolescent health : official publication of the Society for Adolescent Medicine. 2006;39(3):305-17.

253. Patel V, Saxena S, Lund C, Thornicroft G, Baingana F, Bolton P, et al. The Lancet Commission on global mental health and sustainable development. Lancet (London, England). 2018;392(10157):1553-98.

254. Patten SB, Adair CE, Williams JV, Brant R, Wang JL, Casebeer A, et al. Assessment of mental health and illness by telephone survey: experience with an Alberta mental health survey. Chronic diseases in Canada. 2006;27(3):99-109.

255. Pedersen CB, Mors O, Bertelsen A, Waltoft BL, Agerbo E, McGrath JJ, et al. A comprehensive nationwide study of the incidence rate and lifetime risk for treated mental disorders. JAMA psychiatry. 2014;71(5):573-81.

256. Peel A. Severe mental illness registers. Nursing standard (Royal College of Nursing (Great Britain) : 1987). 2005;19(18):38-42.

257. Percudani M, Barbui C, Beecham J, Knapp M. Routine outcome monitoring in clinical practice: service and non-service costs of psychiatric patients attending a Community Mental Health Centre in Italy. European psychiatry : the journal of the Association of European Psychiatrists. 2004;19(8):469-77.

258. Percudani M, Gerzeli S, Massagrandi R, Jommi C, Fattore G, Cerati G, et al. [Costs and outcome of care in subjects with severe mental disorders]. Epidemiologia e psichiatria sociale. 2003;12(3):175-86.

259. Perou R, Bitsko RH, Blumberg SJ, Pastor P, Ghandour RM, Gfroerer JC, et al. Mental health surveillance among children--United States, 2005-2011. MMWR supplements. 2013;62(2):1-35.

260. Picardi A, Lega I, Candini V, Dagani J, Iozzino L, de Girolamo G. Monitoring and evaluating the Italian mental health system: the "Progetto Residenze" study and beyond. The Journal of nervous and mental disease. 2014;202(6):451-9.

261. Picardi A, Tarolla E, de Girolamo G, Gigantesco A, Neri G, Rossi E, et al. [Evaluating the activity of the Italian Mental Health Services inpatient and residential facilities: the PRISM (Process Indicator System for Mental health) indicators]. Rivista di psichiatria. 2014;49(6):265-72.

262. Pirkola S, Saarni S, Suvisaari J, Elovainio M, Partonen T, Aalto AM, et al. General health and quality-of-life measures in active, recent, and comorbid mental disorders: a population-based health 2000 study. Comprehensive psychiatry. 2009;50(2):108-14.

263. Pirraglia PA, Hampton JM, Rosen AB, Witt WP. Psychological distress and trends in healthcare expenditures and outpatient healthcare. The American journal of managed care. 2011;17(5):319-28.

264. Pohjanoksa-Mantyla M, Bell JS, Helakorpi S, Narhi U, Pelkonen A, Airaksinen MS. Is the Internet replacing health professionals? A population survey on sources of medicines information among people with mental disorders. Social psychiatry and psychiatric epidemiology. 2011;46(5):373-9.

265. Public Health Agency of Canada, editor. Report from the Canadian chronic disease surveillance system: mental illness in Canada. Ottawa: Public Health Agency of Canada; 2015.

266. Public Health Agency of Canada, editor. Report from the Canadian chronic disease surveillance system: mood and anxiety disorders in Canada. Ottawa: Public Health Agency of Canada; 2016.

267. Public Health Agency of Canada. Suicide surveillance indicators: Quick Stats, Canada, 2017 edition. Ottawa: Public Health Agency of Canada; 2017.

268. Public Health Agency of Canada. Canadian Chronic Disease Indicators, Quick Stats, 2018 Edition. Ottawa: Public Health Agency of Canada; 2018.

269. Public Health Agency of Canada. Centre for Chronic Disease Prevention. Positive Mental Health Surveillance Indicator Framework: Quick Statistics, adults (18 years of age and older), Canada, 2016 Edition. Ottawa: Public Health Agency of Canada; 2016.

270. Puig-Barrachina V, Malmusi D, Martenez JM, Benach J. Monitoring social determinants of health inequalities: the impact of unemployment among vulnerable groups. International journal of health services : planning, administration, evaluation. 2011;41(3):459-82.

271. Puras D. Mental health in Lithuania. Bulletin of the Board of International Affairs of the Royal College of Psychiatrists. 2005;10.

272. Purebl G, Petrea I, Shields L, Tóth MD, Székely A, Kurimay T, et al. Joint Action on Mental Health and Well-being. Depression, Suicide Prevention and E-Health. Situation analysis and recommendations for action. 2015.

273. Puschner B, Becker T, Bauer S. Routine outcome measures in Germany. International review of psychiatry (Abingdon, England). 2015;27(4):329-37.

274. Rajmil L, Lopez-Aguila S, Mompart Penina A, Medina Bustos A, Rodriguez Sanz M, Brugulat Guiteras P. [Socio-economic inequalities in children's mental health in Catalonia]. Anales de pediatria (Barcelona, Spain : 2003). 2010;73(5):233-40.

275. Rantakokko M, Keskinen KE, Kokko K, Portegijs E. Nature diversity and well-being in old age. Aging clinical and experimental research. 2018;30(5):527-32.

276. Ravens-Sieberer U. The contribution of the BELLA study in filling the gap of knowledge on mental health and well-being in children and adolescents in Germany. European child & adolescent psychiatry. 2008;17 Suppl 1:5-9.

277. Ravens-Sieberer U, Devine J, Bevans K, Riley AW, Moon J, Salsman JM, et al. Subjective well-being measures for children were developed within the PROMIS project: presentation of first results. Journal of clinical epidemiology. 2014;67(2):207-18.

278. Ravens-Sieberer U, Schulte-Markwort M, Bettge S, Barkmann C. [Risks and resources for mental health of children and adolescents]. Gesundheitswesen (Bundesverband der Arzte des Offentlichen Gesundheitsdienstes (Germany)). 2002;64 Suppl 1:S88-94.

279. Reavley NJ, Morgan AJ, Jorm AF. Disclosure of mental health problems: findings from an Australian national survey. Epidemiology and psychiatric sciences. 2018;27(4):346-56.

280. Reeves WC, Strine TW, Pratt LA, Thompson W, Ahluwalia I, Dhingra SS, et al. Mental illness surveillance among adults in the United States. MMWR supplements. 2011;60(3):1-29.

281. Reijneveld SA, Brugman E, Verhulst FC, Verloove-Vanhorick SP. Identification and management of psychosocial problems among toddlers in Dutch preventive child health care. Archives of pediatrics & adolescent medicine. 2004;158(8):811-7.

282. Reilly S, Olier I, Planner C, Doran T, Reeves D, Ashcroft DM, et al. Inequalities in physical comorbidity: a longitudinal comparative cohort study of people with severe mental illness in the UK. BMJ open. 2015;5(12):e009010.

283. Riedel-Heller S, Bramesfeld A, Roick C, Becker T, Konig HH. [Call for more health services research]. Psychiatr Prax. 2008;35(4):157-9.

284. Riley WT, Pilkonis P, Cella D. Application of the National Institutes of Health Patient-reported Outcome Measurement Information System (PROMIS) to mental health research. The journal of mental health policy and economics. 2011;14(4):201-8.

285. Robson D, Gray R. Serious mental illness and physical health problems: a discussion paper. International journal of nursing studies. 2007;44(3):457-66.

286. Roe D, Gelkopf M, Gornemann MI, Baloush-Kleinman V, Shadmi E. Implementing routine outcome measurement in psychiatric rehabilitation services in Israel. International review of psychiatry (Abingdon, England). 2015;27(4):345-53.

287. Roll JM, Kennedy J, Tran M, Howell D. Disparities in unmet need for mental health services in the United States, 1997-2010. Psychiatric services (Washington, DC). 2013;64(1):80-2.

288. Rommel A. [Migration and rehabilitation of mental diseases -- perspectives and limitations in the reporting of official data of service providers]. Gesundheitswesen (Bundesverband der Arzte des Offentlichen Gesundheitsdienstes (Germany)). 2005;67(4):280-8.

289. Roßbach G, Weinbrenner S, Brüggemann S, Märtin S, Rose A. Die Bedeutung psychischer Erkrankungen aus der Perspektive der Deutschen Rentenversicherung. RV aktuell. 2015;62(5/6):114-24.

290. Rothon C, Goodwin L, Stansfeld S. Family social support, community "social capital" and adolescents' mental health and educational outcomes: a longitudinal study in England. Social psychiatry and psychiatric epidemiology. 2012;47(5):697-709.

291. Rüesch P, Manzoni P. Psychische Gesundheit in der Schweiz – Monitoring. Meyer K, Camenzind P, Meyer PC, editors. Zürich: edition obsan; 2003.

292. Ruseski JE, Humphreys BR, Hallman K, Wicker P, Breuer C. Sport participation and subjective well-being: instrumental variable results from German survey data. Journal of physical activity & health. 2014;11(2):396-403.

293. Sachverständigenrat zur Begutachtung der Entwicklung im Gesundheitswesen. Bedarfsgerechte Steuerung der Gesundheitsversorgung. Kapitel 16: Koordinierte Versorgung von Menschen mit psychischen Erkrankungen (S. 683-758). Verfügbar unter: <https://www.svr-gesundheit.de/fileadmin/user_upload/Gutachten/2018/SVR-Gutachten_2018_WEBSEITE.pdf> Zugegriffen: 13. Juli 20182018.

294. Sareen J, Cox BJ, Afifi TO, Clara I, Yu BN. Perceived need for mental health treatment in a nationally representative Canadian sample. Canadian journal of psychiatry Revue canadienne de psychiatrie. 2005;50(10):643-51.

295. Sareen J, Stein MB, Campbell DW, Hassard T, Menec V. The relation between perceived need for mental health treatment, DSM diagnosis, and quality of life: a Canadian population-based survey. Canadian journal of psychiatry Revue canadienne de psychiatrie. 2005;50(2):87-94.

296. Saunders RC, Heflinger CA. Access to and patterns of use of behavioral health services among children and adolescents in TennCare. Psychiatric services (Washington, DC). 2003;54(10):1364-71.

297. Saxena S, van Ommeren M, Lora A, Saraceno B. Monitoring of mental health systems and services: comparison of four existing indicator schemes. Social psychiatry and psychiatric epidemiology. 2006;41(6):488-97.

298. Schibalski JV, Muller M, Ajdacic-Gross V, Vetter S, Rodgers S, Oexle N, et al. Stigma-related stress, shame and avoidant coping reactions among members of the general population with elevated symptom levels. Comprehensive psychiatry. 2017;74:224-30.

299. Schubert CR, Cruickshanks KJ, Dalton DS, Klein BE, Klein R, Nondahl DM. Prevalence of sleep problems and quality of life in an older population. Sleep. 2002;25(8):889-93.

300. Schulenberg JE, Bryant AL, O'Malley PM. Taking hold of some kind of life: how developmental tasks relate to trajectories of well-being during the transition to adulthood. Development and psychopathology. 2004;16(4):1119-40.

301. Scott D, Paterson JL, Happell B. Poor sleep quality in Australian adults with comorbid psychological distress and physical illness. Behavioral sleep medicine. 2014;12(4):331-41.

302. Shiue I. Neighborhood epidemiological monitoring and adult mental health: European Quality of Life Survey, 2007-2012. Environmental science and pollution research international. 2015;22(8):6095-103.

303. Signorini G, Singh SP, Boricevic-Marsanic V, Dieleman G, Dodig-Curkovic K, Franic T, et al. Architecture and functioning of child and adolescent mental health services: a 28-country survey in Europe. The lancet Psychiatry. 2017;4(9):715-24.

304. Sims G, Delaney KR. Implementation of a Health Indicator Monitoring System in an Assertive Community Treatment Team at a Community Outpatient Behavioral Health Organization. Issues in mental health nursing. 2017;38(3):212-8.

305. Siponen U, Valimaki M, Kaivosoja M, Marttunen M, Kaltiala-Heino R. Increase in involuntary psychiatric treatment and child welfare placements in Finland 1996-2003. A nationwide register study. Social psychiatry and psychiatric epidemiology. 2007;42(2):146-52.

306. Skapinakis P, Lewis G, Meltzer H. Clarifying the relationship between unexplained chronic fatigue and psychiatric morbidity: results from a community survey in Great Britain. International review of psychiatry (Abingdon, England). 2003;15(1-2):57-64.

307. Skovgaard AM. Mental health problems and psychopathology in infancy and early childhood. An epidemiological study. Danish medical bulletin. 2010;57(10):B4193.

308. Skovgaard AM, Olsen EM, Christiansen E, Houmann T, Landorph SL, Jorgensen T. Predictors (0-10 months) of psychopathology at age 11/2 years - a general population study in The Copenhagen Child Cohort CCC 2000. Journal of child psychology and psychiatry, and allied disciplines. 2008;49(5):553-62.

309. Skrabski A, Kopp M, Rozsa S, Rethelyi J, Rahe RH. Life meaning: an important correlate of health in the Hungarian population. International journal of behavioral medicine. 2005;12(2):78-85.

310. Slabaugh SL, Shah M, Zack M, Happe L, Cordier T, Havens E, et al. Leveraging Health-Related Quality of Life in Population Health Management: The Case for Healthy Days. Population health management. 2017;20(1):13-22.

311. Smith P, Frank J, Mustard C. The monitoring and surveillance of the psychosocial work environment in Canada: a forgotten determinant of health. Canadian journal of public health = Revue canadienne de sante publique. 2008;99(6):475-7.

312. Smith TE, Appel A, Donahue SA, Essock SM, Jackson CT, Karpati A, et al. Use of administrative data to identify potential service gaps for individuals with serious mental illness. Psychiatric services (Washington, DC). 2011;62(9):1094-7.

313. Sowislo JF, Gonet-Wirz F, Borgwardt S, Lang UE, Huber CG. Perceived Dangerousness as Related to Psychiatric Symptoms and Psychiatric Service Use - a Vignette Based Representative Population Survey. Scientific reports. 2017;8:45716.

314. Pressemitteilung. Ganz normal für die gesetzlichen Kassen: Über 14 Millionen Therapiestunden pro Jahr für die psychische Gesundheit der Versicherten [press release]. 2011.

315. Statistisches Bundesamt (Destatis). Diagnosedaten der Patienten und Patientinnen in Krankenhäusern (einschl. Sterbe- und Stundenfälle). <https://www.destatis.de/DE/Publikationen/Thematisch/Gesundheit/Krankenhaeuser/DiagnosedatenKrankenhaus2120621167004.pdf?__blob=publicationFile>. Zugegriffen: 19. Juli 20182018.

316. Stork E, Scholle S, Greeno C, Copeland VC, Kelleher K. Monitoring and enforcing cultural competence in Medicaid managed behavioral health care. Mental health services research. 2001;3(3):169-77.

317. Strine TW, Balluz L, Chapman DP, Moriarty DG, Owens M, Mokdad AH. Risk behaviors and healthcare coverage among adults by frequent mental distress status, 2001. American journal of preventive medicine. 2004;26(3):213-6.

318. Strine TW, Chapman DP. Associations of frequent sleep insufficiency with health-related quality of life and health behaviors. Sleep medicine. 2005;6(1):23-7.

319. Strine TW, Dhingra SS, Okoro CA, Zack MM, Balluz LS, Berry JT, et al. State-based differences in the prevalence and characteristics of untreated persons with serious psychological distress. International journal of public health. 2009;54 Suppl 1:9-15.

320. Stuart H, Patten SB, Koller M, Modgill G, Liinamaa T. Stigma in Canada: results from a rapid response survey. Canadian journal of psychiatry Revue canadienne de psychiatrie. 2014;59(10 Suppl 1):S27-33.

321. Sundmacher L, Fischbach D, Schuettig W, Naumann C, Augustin U, Faisst C. Which hospitalisations are ambulatory care-sensitive, to what degree, and how could the rates be reduced? Results of a group consensus study in Germany. Health policy (Amsterdam, Netherlands). 2015;119(11):1415-23.

322. Surko M, Pasti LW, Whitlock J, Benson DA. Selecting statewide youth development outcome indicators. Journal of public health management and practice : JPHMP. 2006;Suppl:S72-8.

323. Sussman MP, Jones SE, Wilson TW, Kann L. The Youth Risk Behavior Surveillance System: updating policy and program applications. The Journal of school health. 2002;72(1):13-7.

324. Svensson AC, Fredlund P, Laflamme L, Hallqvist J, Alfredsson L, Ekbom A, et al. Cohort profile: The Stockholm Public Health Cohort. International journal of epidemiology. 2013;42(5):1263-72.

325. Taylor AW, Wilson DH, Dal Grande E, Ben-Tovim D, Elzinga RH, Goldney RD, et al. Mental health status of the South Australian population. Australian and New Zealand journal of public health. 2000;24(1):29-34.

326. Taylor C. Developments in child health surveillance programmes. Nursing times. 2005;101(27):32-4.

327. Teich JL. Monitoring change in behavioral health care. The role of the Center for Mental Health Services. The Psychiatric clinics of North America. 2000;23(2):297-308, vi-vii.

328. ten Have M, Vollebergh W, Bijl RV, de Graaf R. Predictors of incident care service utilisation for mental health problems in the Dutch general population. Social psychiatry and psychiatric epidemiology. 2001;36(3):141-9.

329. The Finnish Institute for Health and Welfare. Statistical information on welfare and health in Finland (formally: Welfare Compass) Helsinki2021 [Available from: <https://sotkanet.fi/sotkanet/en/index>.

330. The ITHACA Project Group. ITHACA Toolkit for Monitoring Human Rights and General Health Care in Mental Health and Social Care Institutions. London: Health Service and Population Research Department, Institute of Psychiatry, King's College London; 2010.

331. Thom J, Bretschneider J, Kraus N, Handerer J, Jacobi F. [Healthcare epidemiology of mental disorders : Why is the prevalence not declining despite growing provision of care?]. Bundesgesundheitsblatt, Gesundheitsforschung, Gesundheitsschutz. 2019;62(2):128-39.

332. TNS Opinion & Social. Special Eurobarometer 248 / Wave 64.4: Mental Well-Being. Brussels: European Commission; 2006.

333. TNS Opinion & Social. Special Eurobarometer 345 / Wave 73.2: Mental Health. Part 1: Report. Brussels: European Commission; 2010.

334. Toffol E, Koponen P, Luoto R, Partonen T. Pubertal timing, menstrual irregularity, and mental health: results of a population-based study. Archives of women's mental health. 2014;17(2):127-35.

335. Topolski TD, Edwards TC, Patrick DL. Toward youth self-report of health and quality of life in population monitoring. Ambulatory pediatrics : the official journal of the Ambulatory Pediatric Association. 2004;4(4 Suppl):387-94.

336. Triguero-Mas M, Donaire-Gonzalez D, Seto E, Valentin A, Martinez D, Smith G, et al. Natural outdoor environments and mental health: Stress as a possible mechanism. Environmental research. 2017;159:629-38.

337. Tsai WL, McHale MR, Jennings V, Marquet O, Hipp JA, Leung YF, et al. Relationships between Characteristics of Urban Green Land Cover and Mental Health in U.S. Metropolitan Areas. International journal of environmental research and public health. 2018;15(2).

338. Tuchsen F, Bach E. Occupation, morbidity, and hospital admission. Scandinavian journal of public health. 2011;39(7 Suppl):141-6.

339. Ungewitter C, Böttger D, Choucair B, El-Jurdi J, Gockel T, Hausner H, et al. Bestandsaufnahme der Versorgung psychisch kranker Menschen in Deutschland: Inanspruchnahmemuster und Kooperation der Leistungserbringer. Abschlussbericht des Forschungsprojektes im Rahmen der Förderinitiative der Bundesärztekammer zur Versorgungsforschung. 2010.

340. Valenstein M, Mitchinson A, Ronis DL, Alexander JA, Duffy SA, Craig TJ, et al. Quality indicators and monitoring of mental health services: what do frontline providers think? The American journal of psychiatry. 2004;161(1):146-53.

341. van Fenema EM. [Assessment of guideline adherence and quality of care with routine outcome monitoring data]. Tijdschrift voor psychiatrie. 2017;59(3):159-65.

342. van Fenema EM, van der Wee NJ, Onstein E, Zitman FG. [Care programmes at mental health centres: the degree of adherence in the first phase of treatment]. Tijdschrift voor psychiatrie. 2010;52(5):299-310.

343. Vasiliadis HM, Lesage A, Adair C, Boyer R. Service use for mental health reasons: cross-provincial differences in rates, determinants, and equity of access. Canadian journal of psychiatry Revue canadienne de psychiatrie. 2005;50(10):614-9.

344. Verow P, Hargreaves C. Healthy workplace indicators: costing reasons for sickness absence within the UK National Health Service. Occupational medicine (Oxford, England). 2000;50(4):251-7.

345. Vreeland B. Bridging the gap between mental and physical health: a multidisciplinary approach. The Journal of clinical psychiatry. 2007;68 Suppl 4:26-33.

346. Waghorn G, Chant D. Overworking among people with psychiatric disorders: results from a large community survey. Journal of occupational rehabilitation. 2012;22(2):252-61.

347. Wahlbeck K. European comparisons between mental health services. Epidemiology and psychiatric sciences. 2011;20(1):15-8.

348. Walter A. Zur Epidemiologie und Versorgungssituation psychischer Erkrankungen. Psych Pflege. 2003;9(06):324-9.

349. Wang J. Mental health treatment dropout and its correlates in a general population sample. Medical care. 2007;45(3):224-9.

350. Wang PS, Aguilar-Gaxiola S, Alonso J, Angermeyer MC, Borges G, Bromet EJ, et al. Use of mental health services for anxiety, mood, and substance disorders in 17 countries in the WHO world mental health surveys. Lancet (London, England). 2007;370(9590):841-50.

351. Watkins KE, Burnam A, Kung FY, Paddock S. A national survey of care for persons with co-occurring mental and substance use disorders. Psychiatric services (Washington, DC). 2001;52(8):1062-8.

352. Webb E, Panico L, Becares L, McMunn A, Kelly Y, Sacker A. The Inter-relationship of Adolescent Unhappiness and Parental Mental Distress. The Journal of adolescent health : official publication of the Society for Adolescent Medicine. 2017;60(2):196-203.

353. Weich S, Sloggett A, Lewis G. Social roles and the gender difference in rates of the common mental disorders in Britain: a 7-year, population-based cohort study. Psychological medicine. 2001;31(6):1055-64.

354. Whisman MA. Marital distress and DSM-IV psychiatric disorders in a population-based national survey. Journal of abnormal psychology. 2007;116(3):638-43.

355. Whitfield J, Jehn L, Kvale K, Grotsky J, Remington P, Jones M. Forward for women's health: the state of women's health in Wisconsin. WMJ : official publication of the State Medical Society of Wisconsin. 2003;102(3):22-8.

356. WHO Regional Office for Europe, editor. Integrated surveillance of noncommunicable diseases (iNCD). A European Union–WHO project. Final project report for the dissemination of results. Copenhagen: WHO; 2015.

357. WHO Regional Office for Europe, editor. Integrated surveillance of noncommunicable diseases. A European Union–WHO project. Copenhagen: WHO; 2015.

358. WHO Regional Office for Europe, editor. Targets and indicators for Health 2020 Version 3. Copenhagen: WHO; 2016.

359. Wijma K, Samelius L, Wingren G, Wijma B. The association between ill-health and abuse: a cross-sectional population based study. Scandinavian journal of psychology. 2007;48(6):567-75.

360. Wittchen H-U. Bedarfsgerechte Versorgung psychischer Störungen. Abschätzungen aufgrund epidemiologischer, bevölkerungsbezogener Daten. 2002.

361. Wittchen H-U, Jacobi F. Die Versorgungssituation psychischer Störungen in Deutschland. Bundesgesundheitsblatt - Gesundheitsforschung - Gesundheitsschutz. 2001;44:993-1000.

362. Wolpert M, Ford T, Trustam E, Law D, Deighton J, Flannery H, et al. Patient-reported outcomes in child and adolescent mental health services (CAMHS): use of idiographic and standardized measures. Journal of mental health (Abingdon, England). 2012;21(2):165-73.

363. World Health Organization, editor. Mental Health Action Plan 2013-2020. Geneva: WHO; 2013.

364. World Health Organization. Mental Health Atlas 2017 Member State Profile - Germany. Geneva: WHO; 2017.

365. World Health Organization, editor. Mental Health Atlas 2017. Geneva: WHO; 2017.

366. World Health Organization. 2018 Global Reference List of 100 core health indicators (plus health-related SDGs). Geneva: WHO; 2018.

367. Xu J, Roberts RE. The power of positive emotions: it's a matter of life or death--subjective well-being and longevity over 28 years in a general population. Health psychology : official journal of the Division of Health Psychology, American Psychological Association. 2010;29(1):9-19.

368. Yang J, Kurdyak P, Guttmann A. Developing Indicators for the Child and Youth Mental Health System in Ontario. Healthcare quarterly (Toronto, Ont). 2016;19(3):6-9.

369. Yoon S. Child maltreatment characteristics as predictors of heterogeneity in internalizing symptom trajectories among children in the child welfare system. Child abuse & neglect. 2017;72:247-57.

370. Zach A, Meyer N, Hendrowarsito L, Kolb S, Bolte G, Nennstiel-Ratzel U, et al. Association of sociodemographic and environmental factors with the mental health status among preschool children-Results from a cross-sectional study in Bavaria, Germany. International journal of hygiene and environmental health. 2016;219(4-5):458-67.

371. Zahran HS, Kobau R, Moriarty DG, Zack MM, Holt J, Donehoo R. Health-related quality of life surveillance--United States, 1993-2002. Morbidity and mortality weekly report Surveillance summaries (Washington, DC : 2002). 2005;54(4):1-35.

372. Zhang W, Chen Q, McCubbin H, McCubbin L, Foley S. Predictors of mental and physical health: individual and neighborhood levels of education, social well-being, and ethnicity. Health & place. 2011;17(1):238-47.

373. Zhao G, Ford ES, Li C, Strine TW, Dhingra S, Berry JT, et al. Serious psychological distress and its associations with body mass index: findings from the 2007 Behavioral Risk Factor Surveillance System. International journal of public health. 2009;54 Suppl 1:30-6.
